# Binary Phase Diagrams and Thermodynamic Properties of Silicon and Essential Doping Elements (Al, As, B, Bi, Ga, In, N, P, Sb and Tl)

**DOI:** 10.3390/ma10060676

**Published:** 2017-06-20

**Authors:** Ahmad Mostafa, Mamoun Medraj

**Affiliations:** 1 Mechanical and Materials Engineering Department, Khalifa University of Science and Technology, Masdar Institute, Masdar City 54224, UAE; amostafa@masdar.ac.ae; 2 Mechanical Engineering Department, Concordia University, 1515 Rue Sainte Catherine West, Montreal, QC H3G 2W1, Canada

**Keywords:** binary phase diagrams, doping diffusion, solar-grade silicon, thermochemical data

## Abstract

Fabrication of solar and electronic silicon wafers involves direct contact between solid, liquid and gas phases at near equilibrium conditions. Understanding of the phase diagrams and thermochemical properties of the Si-dopant binary systems is essential for providing processing conditions and for understanding the phase formation and transformation. In this work, ten Si-based binary phase diagrams, including Si with group IIIA elements (Al, B, Ga, In and Tl) and with group VA elements (As, Bi, N, P and Sb), have been reviewed. Each of these systems has been critically discussed on both aspects of phase diagram and thermodynamic properties. The available experimental data and thermodynamic parameters in the literature have been summarized and assessed thoroughly to provide consistent understanding of each system. Some systems were re-calculated to obtain a combination of the best evaluated phase diagram and a set of optimized thermodynamic parameters. As doping levels of solar and electronic silicon are of high technological importance, diffusion data has been presented to serve as a useful reference on the properties, behavior and quantities of metal impurities in silicon. This paper is meant to bridge the theoretical understanding of phase diagrams with the research and development of solar-grade silicon production, relying on the available information in the literature and our own analysis.

## 1. Introduction

The primary energy sources for electrical power generation are recognized as unsustainable. For instance, combustion of fossil fuels (coal, petroleum, and natural gas) emit massive quantities of CO_2_ in the atmosphere that leads to severe climate changes, such as global warming, sea level rise and change in the rain fall patterns [1,2,3]. Nuclear power plants are more environmentally friendly (emit zero CO_2_. However, these plants can be extremely dangerous, if unsafely operated as was the case in the Chernobyl disaster in 1986 [4], or if natural disasters take place in events such as the Fukushima nuclear accident [5]. Storage of the highly radioactive waste is another serious issue that must be taken into consideration [1]. It is expected that both safety and high-level nuclear waste issues will be more seriously examined in the next generation of nuclear power reactors. For the reasons mentioned above, more use of clean and renewable energy sources is crucial. Sources of renewable energy, solar in particular, can provide vital solutions to problems associated with fuel combustion and nuclear fission. The solar energy is transferred into direct current electrical power via the photovoltaic (PV) effect [6], which was discovered and demonstrated by the Nobel Laureate Becquerel [7,8].

Solar cells are traditionally divided into three different generations, these are: silicon-based solar cells, thin-films solar cells and solar cells based on nano-crystals and nano-porous materials [2,3,6,9]. Most of the available solar cell modules for contemporary market demands are made of crystalline silicon (c-Si). Thin film solar cells (TFSC) [10,11], including: amorphous silicon (a-Si), copper indium gallium diselenide Cu(In,Ga)Se_2_ (CIGS), cadmium telluride (CdTe) and others, represent 10% of the solar cells market [1]. The third generation of solar cells, include dye-sensitized solar cells (DSSC), hybrid organic solar cells and quantum dot solar cells, and are still under research and development [1,3,9].

The PV industry is currently undergoing significant development. It is difficult to predict which technical pattern has to be followed to achieve the desired outcomes from PV devices. It is important to know which materials will be utilized and what conditions are suitable to ensure maximum efficiency and long-term energy provision. Photovoltaic efficiency is highly influenced by temperature, dopant amount and density of imperfections [1,12]. These conditions might be optimized using heuristic approaches. However, it will be a very long journey to design a perfect PV system. The major goal in solar cell fabrication is to design a process that can minimize the negative effects of impurities or at least passivate them through a better understanding of their relationships with silicon. In this work, we use our knowledge of phase diagrams, obtained by investigating several alloying systems, to understand basic concepts for improvement in PV device performance while also providing thermodynamic properties of the Si-based binary systems. In the course of this work, we focus on crystalline silicon solar cells and binary phase diagrams of silicon with different doping elements, such as Al, As, B, Bi, Ga, In, N, P, Sb and Tl. This should serve as a useful reference for the properties, behavior and quantities of metal impurities in silicon. It is worth mentioning that this work will not provide a theoretical background on c-Si solar cell fabrication or discussion associated to energy levels of semiconductors, but rather serves as a guideline to control the processing parameters of solar and electronic Si through understanding the phase diagram, thermodynamic and diffusion data.

## 2. Crystalline Silicon Solar Cells

Although they have a theoretical efficiency limit of about 26% [12,13], due to the presence of surface recombination that depletes the minority carriers [14,15,16], crystalline silicon solar cells have dominated PV modules [17]. These cells constitute more than 85% of the worldwide PV market [1]. Dominance of c-Si in PV technology stems to a great extent from the development of Si for the microelectronic applications [18,19], besides its relatively low manufacturing cost, non-toxicity and availability [1,13,15,20]. Both laboratory and industrial-type crystalline silicon solar cells are divided into monocrystalline Si, block-cast polycrystalline Si, ribbon Si and thin-film polycrystalline Si according to the type of the starting silicon wafer [1,2,9,13,15]. 

Crystalline silicon is an indirect semiconductor and its electrical properties deteriorate largely by the level of defects (bulk and/or surface defects), because they form numerous clusters that capture charge carriers. These defects are classified as intrinsic and extrinsic [15]. Intrinsic defects in pure silicon can be vacancies or self-interstitials that play an important role in many defect processes, such as self or dopant diffusion, strain release in the lattice and radiation defects [17]. Extrinsic defects can be doping atoms, transition metals, interstitial oxygen atoms or carbon substitutional atoms [17,18,21]. The following will be an attempt to summarize a general understanding of impurities in silicon.

## 3. Impurity Atoms

The concentration space of impurity atoms in the solar and electronic Si ranges from particle per billion (ppb), which represents a challenge to measure for some impurities [22], to few percentages. However, a small change in the impurity level of a PV junction can result in an unacceptable drop in performance. Impurities exist in the Si lattice, as substitutional or interstitial point defects, in the form of precipitates or at the surfaces of the silicon wafers. Their distribution depends on the solubility limits and diffusivity as functions of atomic size and affinity to form bonds with silicon atoms [23]. 

Solubility limits can be directly determined from a well-established phase diagram as a function of temperature. Processing temperatures can be optimized with the help of a phase diagram to achieve the desired concentration level of impurity atoms. 

**Figure 1:**
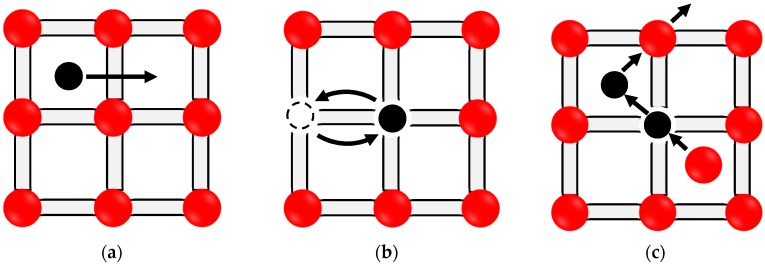
(**a**) Interstitial, (**b**) Vacancy and (**c**) interstitialcy diffusion mechanisms.

Knowledge of diffusion mechanisms and diffusivity of impurity atoms is essential to understand the microstructural changes in solar and electronic silicon at any temperature, which can be supported by phase equilibrium studies. Three possible mechanisms of atomic diffusion were established [24,25]. These are: (1) the interstitial mechanism, where the small radius impurity can move from one interstitial position to another, such as group IA and group VIIIA elements; (2) the vacancy mechanism, where substitutional impurity moves via neighboring vacant sites through the host lattice; (3) interstitialcy mechanism (combined interstitial and vacancy mechanisms), where the diffusion occurs when a substitutional impurity resides at an interstitial site replacing a silicon atomand the replaced Si atom undergoes self-interstitial within the regular lattice site [26]. The three diffusion mechanisms are illustrated in Figure 1.

The diffusivity of impurity atoms depends exponentially on temperature according to Arrhenius Equation (1), as follows:

(1) $$D=D_{0}\exp\left(-\frac{Q_{d}}{RT}\right){\mathrm{cm}}^{2}/s$$

where, *Q_d_* is the activation energy and *D*_0_ is the temperature-independent pre-exponential factor. *R* and *T* are the universal gas constant and the absolute temperature. Impurity with high diffusivity tends to move toward nucleation sites and gives rise to structure relaxation [23].

The success of producing or refining Si materials to high purity levels depends heavily on the availability and reliability of phase diagrams, thermodynamic and diffusion data. This information act as an important tool in evaluating the effects of impurities on the phase equilibria in solar and electronic Si systems. Many impurity atoms may exist in Si wafers, such as oxygen, carbon, transition metals, alkali and alkali-earth impurities and groups IIIA and VA metals [27]. However, in this work, we focus on the impurity atoms of group IIIA (*p*-type) and group VA (*n*-type).

## 4. Impurity Atoms from Groups IIIA and VA

Atoms of these groups act as substitutional impurities in Si. When an atom from group IIIA replaces a Si atom, the remaining valence electrons are insufficient to satisfy the four covalent neighboring bonds. This gives rise to the formation of holes that are weakly tied to group IIIA atoms. Thus, group IIIA atoms are acceptors. On the other hand, group VA impurity atoms are called donors, because they replace a Si atom and one electron remains untied to group VA atom. This extra electron can be easily activated and sent to the conduction band [24]. Besides their suitability as donors and/or acceptors, the binary phase diagrams of Al, As, B, Bi, Ga, In, N, P, Sb and Tl with Si exhibit some similarities. The main similarity between some of these phase diagrams is that the maximum solubility of an impurity atom occurs above the eutectic temperature. This is due to the high entropy of the disordered state, which results in forming defects such as vacancies [28]. This behavior is called retrograde or inverse solubility [28,29]. The retrograde solubility phenomenon occurs during solidification and originates from a miscibility gap in the free energy of mixing [30]. Impurity concentration in the melt varies with the fraction of melt solidified and the value of the impurity distribution coefficient, *K_o_*, which is also known as the segregation or partition coefficient [31]. *K_o_* of group IIIA and group VA dopants in Si is less than one, as listed in Table 1 [28,32]. When *K_o_* is less than one, impurities accumulate progressively at the liquid/solid interface during the continuous crystal pulling process. These impurities deplete when *K_o_* is greater than one [28]. This provides insight on the technical challenges related to producing large volume Si substrates with uniform dopant distribution. From a refining point of view, impurities with a very low distribution coefficient can easily be removed since they accumulate at the bottom end of the crystal and contamination does not occur. For those having higher distribution coefficients (close to unity), they remain in the melt and accumulate in the upper end of the crystal. Thus, some separate chemical processes or multiple zone melting are required to clean them up [33].

**Figure 2:**
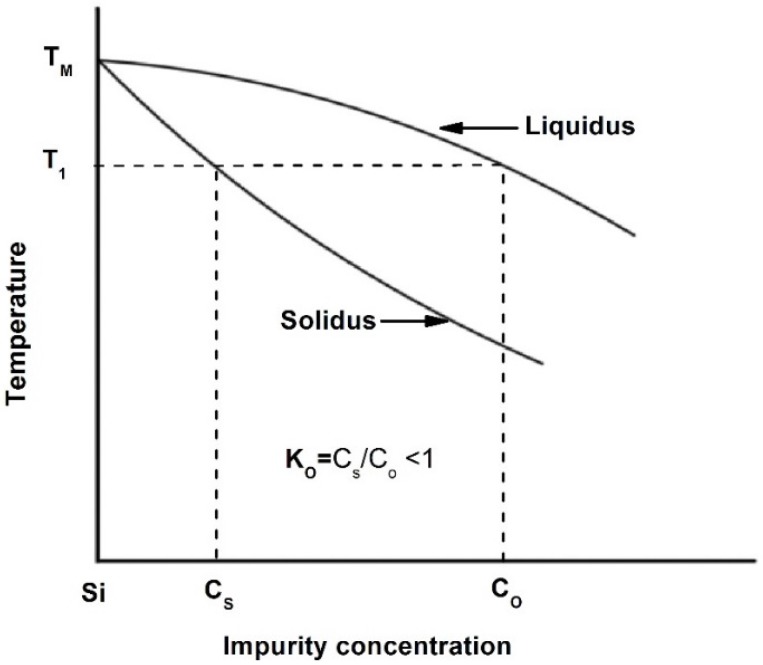
Determination of the distribution coefficient using a phase diagram.

The equilibrium distribution coefficient is the ratio of the impurity concentration in the solid, *C_s_*, to that in the liquid, *C_o_*, at a given temperature, e.g., T_1_ in Figure 2. *K_o_* can be obtained from a phase diagram as demonstrated in Figure 2.

From a practical point of view, *K_o_* is used to find the maximum molar solid solubility (*X_m_*) of impurities in silicon through the empirical relationship [34] given in Equation (2):

(2) $$X_{m}=0.1\times K_{o}$$

When the unit of atom/cm^3^ is used for the maximum solid solubility, *C_M_*, Equation (2) can be re-written as in Equation (3) [34]:

(3) $$C_{M}=\left(5.2\times 10^{21}\right)\times K_{o}$$

This relation was extracted from the maximum solid solubility vs. distribution coefficient plot of the following group of elements: Fe, Zn, S, Mn, Au, Cu, Al, Ga, Li, Sn, Sb, As and P. Although it is not exact for some elements, it may be useful to predict one quantity, either *C_m_* or *K_o_*, if the other one is known, or to evaluate some experimental data [34]. For the aforementioned similarities, phase diagrams of groups IIIA and V elements are evaluated giving special attention to the Si-rich terminal side, due to its practical relevance to production of solar and electronic Si.

## 5. Phase Diagrams

One of the major reasons to analyze Si binary phase diagrams is that doping elements have a steep temperature-dependent solid solubility limit that renders Si easily supersaturated with them upon cooling. Therefore, they tend to form crystal defects in form of complex precipitates at grain boundaries [1]. These defects are not preferable since they deteriorate solar cell efficiency. 

Knowledge of the phase equilibrium and thermodynamic properties of the Si-impurity binary phase diagrams is important for understanding and improving the refining process of metallurgical Si grade feedstock to solar cell grade. For instance, the solvent refining process is one of the metallurgical approaches used for solar-grade silicon production, especially when impurity precipitates exhibit very limited solubility in silicon [35]. In this methodology, the silicon alloy is kept at a temperature above the liquidus, and then the temperature is reduced to a value near the eutectic temperature. As a result, purification solidification takes place and leaves impurities in the liquid phase. This means that purification occurs due to impurity rejection by the solidification front [36]. The success of the solvent refining process depends on the Si-terminal solubility which is decided by careful examination of the solvus line of the equilibrium phase diagram. The accuracy of the solvus lines in an equilibrium phase diagram is highly dependent on the solubility limit measurements. If the solubility values of specific impurities for a specific system are unknown and/or difficult to determine experimentally, or their behavior cannot be extrapolated, thermodynamic modeling is used [37].

Solidification process parameters such as the composition of the alloy, temperature and influence of cooling rate on the microstructure controls the impurity level of the crystallized silicon and can be determined from a well-established phase diagram. In this work, binary phase diagrams of Si with *p*-type elements (Si-{B, Al, Ga, In, Tl}) and Si with *n*-type elements (Si-{N, P, As, Sb, Bi}) have been assessed based on the available experimental data and thermodynamic descriptions. Throughout the paper, solid blue color lines are used in the figures to represent our best understanding of the phase diagram and thermodynamic data. All data points, obtained from the literature, are presented in phase diagram and thermodynamic properties figures in order to illustrate the error range. Error bars are provided whenever they are available in the literature. The phase diagrams and thermodynamic properties plots are in mole fraction; whereas, concentrations of impurity elements in the text are in atomic percentage. The crystallographic data for solid phases of all systems are summarized in Table 2. This table will be referred to during the discussion of each binary system.

### 5.1. The Al-Si System

**Figure 3:**
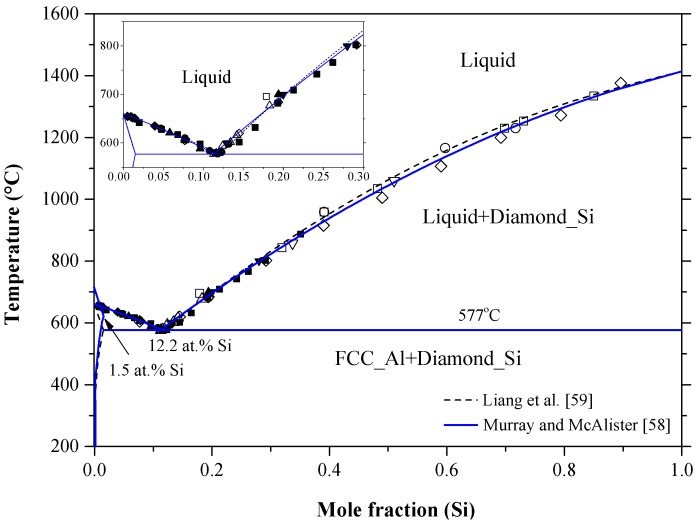
The Al-Si phase diagram redrawn after [58]; -----: [59]; **+**: [65]; ○: [76]; □: [77]; Δ: [78]; ▽: [79]; ◇: [80]; ●: [81]; ■: [82]; ▲: [83,84]; ▼: [85]; ♦: [86].

**Figure 4:**
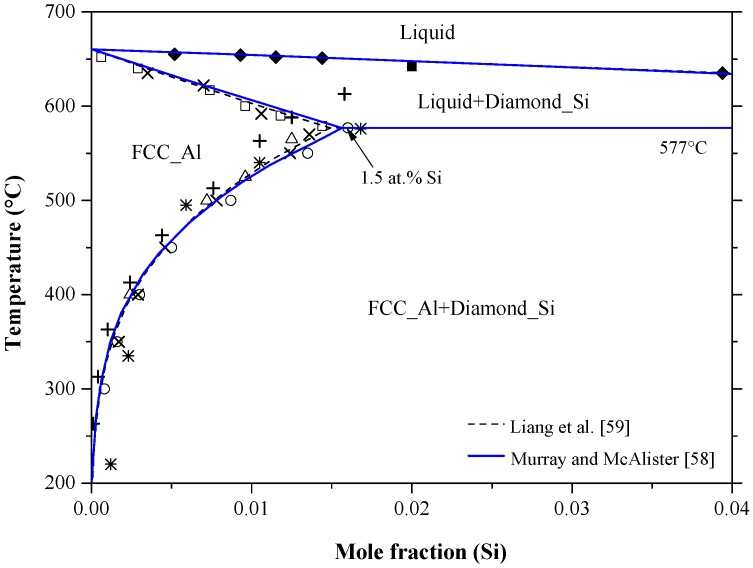
Al-rich side of the Al-Si phase diagram [58]; ----: [59]; ×: [60]; *: [61]; ○: [62]; □: [63]; Δ: [64]; +: [65].

**Figure 5:**
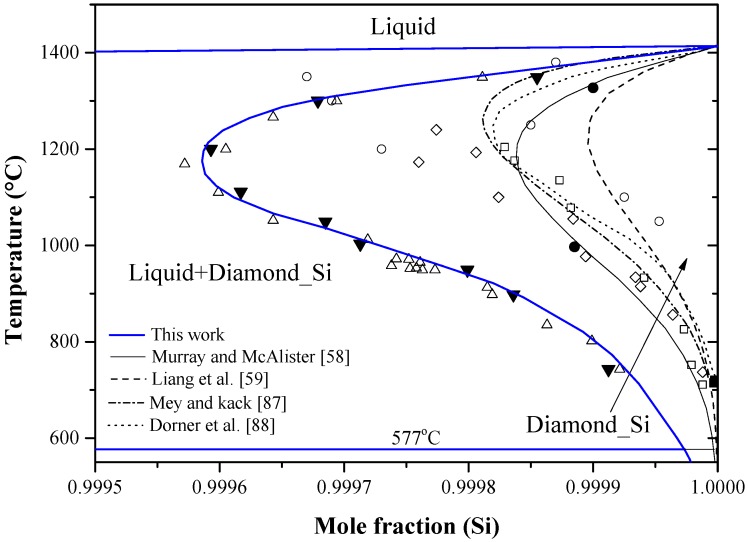
Magnified part of the Si-rich side of the Al-Si phase diagram; ―: [58]; -----: [59]; -·-·-: [87]; ····: [88]; ■: [29]; ●: [65]; □: [68]; ○: [69]; Δ (EPMA): [70], ▼ (Hall measurements): [70]; ◇: [89].

The Al-Si phase diagram in Figure 3 was redrawn after Murray and McAlister [58] based on the available experimental and thermodynamic data in the literature. The dashed line represents the calculated system by Liang et al. [59]. The system exhibits a eutectic reaction and two terminal solid solutions. The eutectic occurs at 12.2 at % Al and 577 °C. Many researchers [60,61,62,63,64,65] studied the solvus line in the Al-rich side as shown in Figure 4. The maximum solid solubility of Si in Al terminal solid solution is about 1.5 at % Si. The liquidus curve and the thermodynamic activity of the melts have been extensively reviewed and recalculated by Safarian et al. [66,67]. The solvus curve of the Si terminal solid solution among the different studies was inconsistent, as can be seen in Figure 5. In addition to the experimental errors, the data scattering could be due to the presence of low concentration impurities, which reduce the accuracy of the measurements. Gudmundsen and Maserjian [29], using thermal gradient experiments and spectrophotometric analysis, measured the solubility of Al in Si as 2.9 × 10^−4^ and 2.4 × 10^−4^ at % at 715 °C and 720 ± 10 °C, respectively, as compared to Mondolfo [65] who reported the solubility of Al in Si to be ranging from 1 × 10^−2^ at % at 1327 °C to 1.15 × 10^−2^ at % at 997 °C. Navon and Chernyshov [68] measured the solubility of Al in Si in the 700–1200 °C temperature range using resistivity measurements. Their measurements showed fair agreement with the data of Miller and Savage [69] at 1200 °C but not at lower temperatures, due to the inaccuracy of diffusion measurements [69] at low temperatures. Navon and Chernyshov [68] further reported a retrograde behavior in the Si-rich side with a maximum of 1.7 × 10^−2^ at % Al at around 1204 °C. In their review, Murray and McAlister [58] estimated the maximum solubility of Al in Si, based on the experimental results of [65,68], to be 1.61 × 10^−2^ at % Al at about 1210 °C. 

In a recent study, Yoshikawa and Morita [70] measured the solid solubility of Al-Si melts, prepared by true gradient zone melting (TGMZ) method, using electron probe microanalyzer (EPMA) and Hall measurements, and reported a similar retrograde solubility behavior to that of [68]. The solubility values obtained from the two measuring methods [70] were consistent with each other and gave maximum at 4.3 × 10^−2^ at % Al and 1177 °C. The same level as the solubility of Al in Si [70] was obtained by Nishi et al. [71] using inductively coupled plasma (ICP spectrometry. Although these values are higher than those in the literature, yet they are accepted in the current work, because Yoshikawa and Morita [70] formed Si single crystal using TGMZ method, which makes the hole mobility measurements possible. The crystallographic data of the terminal solid solutions in Al-Si system are listed in Table 2.

It is worth mentioning that silicon films formed from Al-Si melt are not suitable as PV materials, because of the high solubility of Al in liquid Si. In other words, the number of aluminum acceptors would increase dramatically upon cooling due to Al supersaturation in Si lattice. The solubility of Al can be lowered by adding other elements to the melt such as P, which reacts with aluminum to form small insoluble particles of AlP intermetallic [72].

It is proven experimentally that the solubility of Si in Al can be increased using rapid quenching process of Al-Si melts under pressure [37]. Soma et al. [73], using first principle calculations, measured the solubility of Si in Al under pressure of 0, 3, 5 and 10 GPa. They concluded that the solid solubility of silicon in aluminum extends from 1.6 to ~30.0 at % under pressure and their results were in good agreement with the available experimental data [74,75]. No information about the Al solubility in Si at high pressure could be found in the literature.

Aluminum is considered the fastest diffusing acceptor dopant in silicon, thus it is used to fabricate deep n-p junctions with large thicknesses [90,91]. Despite their industrial importance, fabrication of aluminum n-p junctions requires high processing temperature of about 1250 °C, corresponding to the maximum Al solubility in Figure 5, and a long time of 40 h [91]. These severe conditions give rise to defect formation and to possible contamination. Furthermore, new processing techniques, such as ion implantation [92], focus on shortening the diffusion time and lowering the diffusion temperature. In order to achieve the optimum processing conditions, the diffusion behavior of Al in Si must be very well understood. The diffusivity of aluminum in silicon was measured by Krause and Ryssel [91] in the 850–1290 °C temperature range. They measured both intrinsic, $D_{i}$, and extrinsic, $D_{ex}$, diffusion coefficients and the results are summarized in Table 3. The diffusivity of several elements in silicon, including those involved in the current work, has been reported in the literature. More details are available in the works of Fisher [93] and Tang et al. [94].

**Figure 6:**
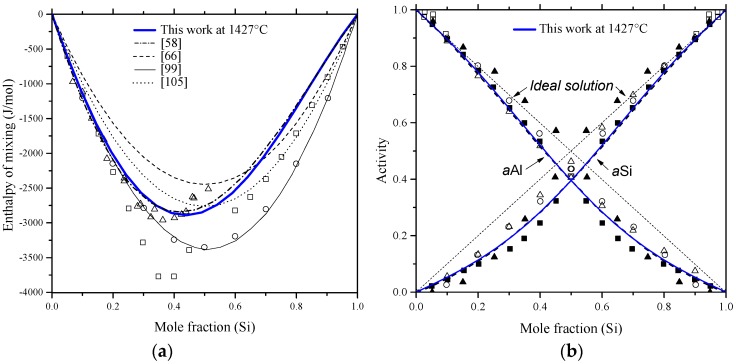
(**a**) Calculated enthalpy of mixing of the Al-Si liquid; ―: [99] at 1600 °C; ○: [104] at 1600 °C; □: [103] at 1547 °C; Δ: [102] at 1427 °C; -·-·-: [58]; -----: [66]; ····· : [105] at 1427 °C and (**b**) Calculated activity of Al and Si in the Al-Si liquid at 1427 °C; -----: [99] at 1600 °C; ○: [106]; □: [107] and Δ: [100] at 1427 °C; ■: [103] at 1547 °C; ▲: [108] at 1700 °C.

Chakraborti and Lukas [95] critically reviewed the available phase diagram and thermodynamic data of Al-Si system in the literature. Using computational thermodynamics, enormous number of Al-Si phase diagram calculations were performed [27,59,87,88,96,97,98,99,100,101]. The thermodynamic properties, enthalpy of mixing and activity, are calculated as shown in Figure 6a,b, respectively. The thermodynamic parameters of the Al-Si system are listed in Table 4.

In Figure 6a, the calculated enthalpy of mixing of the liquid phase by Murray and McAlister [58] at 1427 °C agree with the reaction calorimetric result of Bros et al. [102] within ±225 J·mol^−1^ only at mid-composition, but differ 900 J·mol^−1^ from the results of Korber et al. [58] and 500 J·mol^−1^ from Gizenko et al. [103]. In contrast, Tang et al. [99] gave higher weight to the experimental results of Rostovtsev and Khitrik at 1600 °C [104]. Other calculations performed by Safarian et al. [66] and Tang et al. [105] were lower than any of the reported values. The low values might be as a result of attempts made by authors [66,105] to be consistent with the phase diagram data only. On the other hand, experimental points given by Gizenko et al. [103] showed higher enthalpy of mixing than others. This might explain why these results were not considered in thermodynamic calculations of [66,99,105]. The calculated enthalpy of mixing in the present work, taking into account all the available data sets, agree with the experimental data of [102] in the 58–100 at % Al range and consistent with the calculations of [58] in the full composition range at 1427 °C. Thus, the calculations made in this work are more reliable for thermodynamic modelling purpose. The calculated activity in Figure 6b agrees very well with the experimental data of [103]. The other data points might be scattered due to the difference in the measurement temperatures. The negative deviation from Raoult’s law indicates that the liquid solution is exothermic.

### 5.2. The As-Si System

**Figure 7:**
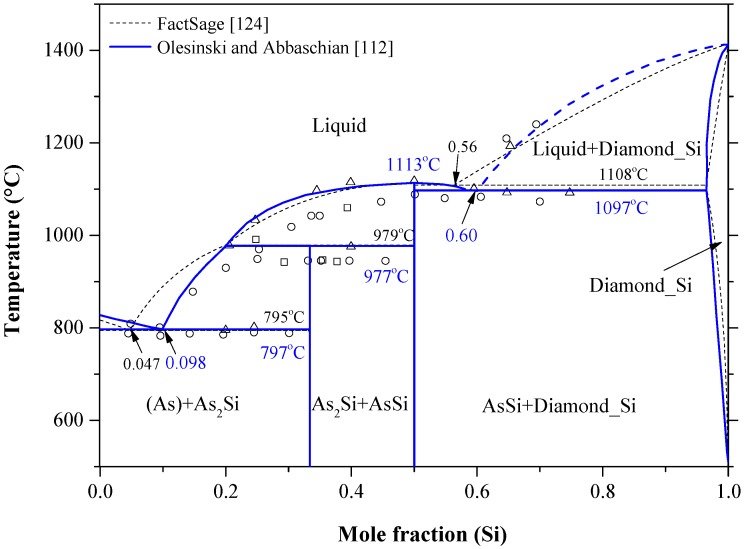
The As-Si phase diagram at ~40 bar [112]; ----: [124]; ○: [113]; □: [114]; Δ: [115].

This phase diagram has been mainly studied under pressure, because the sublimation temperature of arsenic at atmospheric pressure is low (614 °C) [109]. The melting under pressure of As occurs at 940 °C and 59.2 atm [110]. Talc was used as a solid pressure-transmitting medium to generate pressure inside the test chamber [111]. The As-Si equilibrium phase diagram at a pressure of ~39.5 atm was evaluated by Olesinski and Abbaschian [112] based on the available experimental data [42,113,114,115,116,117,118,119] and thermodynamic descriptions [120,121,122,123], as shown in Figure 7. The liquid phase boundary of the As-Si system in the Si-rich side, up to the eutectic point at around 56 at % Si, was drawn as dotted line in the work of Olesinski and Abbaschian [112] due to the large disagreement among the available data [113,115]. However, the liquidus in the As-rich side and solidus lines agree very well with the results of Ugay et al. [115], who performed vapor pressure measurements using a static manometric method and provided results with higher accuracy than those obtained by Klemm and Prscher [113] and Ugay et al. [114]. The system includes two intermetallic compounds and two terminal solid solutions. The AsSi intermetallic compound melts congruently at 1113 °C; while As_2_Si undergoes peritectic transformation at 977 °C. The crystallographic data of the solid phases in the As-Si system are given in Table 2. The data of Beck [40] and Wadsten et al. [42] for the AsSi (monoclinic crystal system, AsSi prototype and *C2/m* space group) and As_2_Si (orthorhombic crystal system, As_2_Ge prototype and *Pbam* space group) phases, respectively, at room temperature are accepted in this work. It is worth mentioning that the stable high-pressure structure of As_2_Si is pyrite-type, which is not achievable under ambient conditions [119].

**Figure 8:**
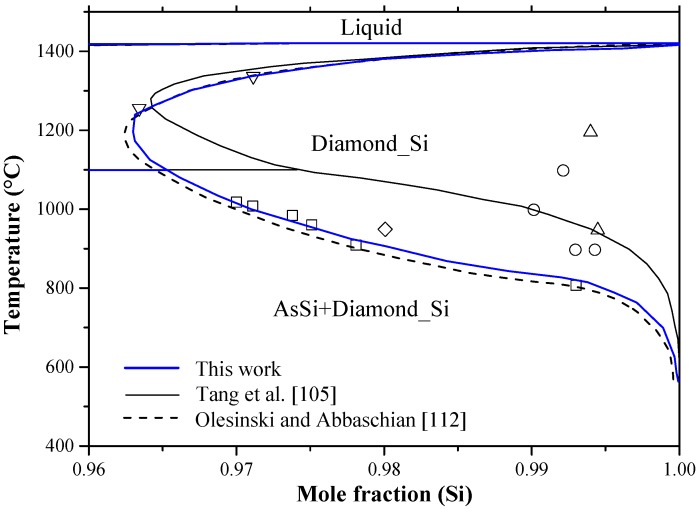
Magnified part of the As-Si phase diagram; —: [105]; ----: [112]; □: [117]; ▽: [118]; ◇: [126]; ○: [130]; Δ: [131].

The solubility of Si in As is considered negligible, whereas solubility of As in Si shows a retrograde behavior in liquid silicon. It can reach to around 3.25 at % at the eutectic temperature. Several research works [118,125,126,127,128,129,130,131] focused on the Si-rich side to determine the solid solubility limits of arsenic in silicon. According to Olesinski and Abbaschian [112], the maximum solubility of As was reported as 3.5 at % As at ~1200 °C based on the work of Sandhu and Reuter [117], who determined the solvus line from the vapor pressure measurements using radiotracer and chemical methods. Other experimental points were excluded, because they were acquired by electrical conductivity methods that are known to give high error of measurements in this system. Tang et al. [105] assessed the As-Si system based on the experimental data of [113,114,117,118,125,126,131] and the thermodynamic activity data of [116,117]. They [105] re-evaluated the maximum solubility of As in Si to be around 3.4 at % As at 1300 °C, favoring the data of [118,130] over [117,126] without providing any justification. The Si terminal solid solution was described [105] based on a substitutional solution with random mixing assumption. Although Tang et al. [105] incorporated several experimental points in their optimization, the assessment of Olesinski and Abbaschian [112] is favorable in this work, because they are self-consistent. Figure 8 summarizes the solid solubility limits of As in Si and the acceptable solvus line in this work, which provides a maximum solubility of 3.52 at % As at 1200 °C.

It is worth mentioning that if As concentration is higher than the solubility limit, the electrical properties of the heavily As-doped Si deteriorates due to the occurrence of electrically inactive arsenic clusters [125,132,133]. The electrically inactive cluster contains two As atoms and one vacancy position [125,134]. Furthermore, these clusters result in obstructing the phase equilibrium between Si and the AsSi compound by blocking the nucleation of AsSi precipitates. Hence, it is of great importance to determine the maximum solubility of As in Si precisely. Nobili and Slomi [134] described the maximum As concentration, *C_sat_*, in Si lattice at equilibrium with AsSi by $C_{sat}=1.3\times 10^{23}e^{\left(-0.42\;eV/kT\right)}\;{\mathrm{cm}}^{-3}$ based on the best fit equation given in Nobili’s earlier work [128] in the 800–1050 °C temperature range. 

Impurity atoms may occupy either substitutional or interstitial locations in Si lattice. However, arsenic shows mixed vacancy-interstitial (i.e., interstitialcy as illustrated in Figure 1c) diffusivity in Si [26,135]. It has almost similar tetrahedral radius as silicon (As = 1.24 Å and Si = 1.18 Å), thus it does not strain the silicon lattice. However, at high arsenic concentration, clusters form and retard the diffusivity of As in Si [136]. Intrinsic diffusivity of As in Si can be expressed as $D_{i}=22.9\times e^{\left(-4.1\;eV/kT\right)}{\mathrm{cm}}^{2}/s$ in the 900–1250 °C temperature range [137]. Arsenic diffusion in Si is quiet complex due to the change of diffusion mechanism depending on As concentration and temperature. Additional information about arsenic diffusion mechanisms can be found in Hull [138] and Fair [137].

The As-Si system was recalculated using FTlite thermodynamic database integrated in FactSage^®^ software (version 7.0, Thermofact and GTT-Technologies, Montreal, Canada) [124]. In some regions, the calculated phase diagram showed reasonable agreement with the experimental results presented by Ugay et al. [115]. However, many discrepancies were detected. Figure 7 shows both versions of the As-Si phase diagram [112,124] and Table 5 summarizes the differences between these two versions. As can be seen from Figure 7 that the liquidus curve presented by [112] is more convincing than that of [124], because it matches better with the experimental points on both composition extremes. Furthermore, due to the retrograde behavior of the Si-rich solvus line, the shape of Si-rich liquidus in [112] is more appropriate when considering the limiting slopes theory [139].

**Figure 9:**
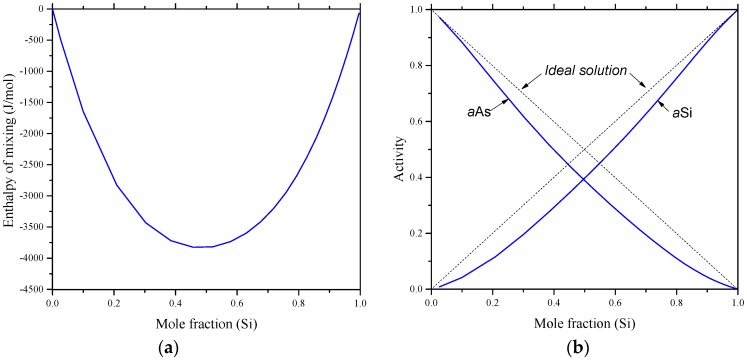
(**a**) Enthalpy of mixing of the As-Si liquid and (**b**) activity of As and Si in the As-Si liquid calculated in this work at 1600 °C using FactSage^®^ software [124].

The thermodynamic properties, enthalpy of mixing and activity of As-Si liquids, are calculated using FTlite database and presented in Figure 9. No experimental enthalpy of mixing or activity of the liquid As-Si alloys could be found in the literature to compare with. The calculated activity curve of liquid As and Si alloys at 1600 °C shows a negative deviation from the ideal behavior. The negative deviation from Raoult’s law could be due to the stronger molecular interaction between As/Si atoms than the interactions between Si/Si and As/As atoms.

The enthalpy of formation of the As-Si intermetallic compounds was determined by Fitzner and Kleppa [140] using direct synthesis drop calorimetry. The actual value of enthalpy of formation of the AsSi compound could be higher than that listed in Table 6, because the measurements were performed on an alloy containing a mixture of AsSi + As_2_Si rather than on a pure AsSi compound. These experimental results were compared with those predicted by Niessen et al. [141] using the semi-empirical theory of. The predicted results are significantly higher than those obtained experimentally by Fitzner and Kleppa [140], as indicated in Table 6. The large negative values of the calculated enthalpies of formation are because the liquid arsenic that should occur under atmospheric pressure was not considered. It is worth mentioning that the liquid arsenic was treated as a solvent in the work of Niessen et al. [141]. Based on our state of knowledge of this system we recommend further experimental efforts to measure the enthalpy of mixing of the liquid solution, the activities in the liquid phase and most importantly the maximum solubility of As in Si. After this is done, a new thermodynamic modeling considering all the new data should be carried out.

### 5.3. The B-Si System

Boron is a primary *p*-type doping element in silicon wafers, with the highest solid solubility in silicon as compared to all elements in group IIIA [90]. According to Zaitsev and Kodentsov [142], the solid solubility of B in Si can reach up to 3.5 at % B at 1362 °C. The B-Si phase diagram was assessed by Olesinski and Abbaschian [143] based on the previous experimental data [118,144,145,146,147] and thermodynamic calculations [148,149,150]. Three intermetallic compounds were reported. Two of them, B_n_Si (14 < *n* < 40) and B_6_Si, form peritectically at 2020 °C and 1850 °C, respectively. Whereas, the third intermetallic compound, B_3_Si, was found to form by a peritectoid reaction at 1270 °C. The crystallographic data of the intermetallic compounds in the B-Si system are listed in Table 2. B_3_Si and B_n_Si were represented as intermediate solid solutions with unknown solubility ranges [143] while B_6_Si was represented as stoichiometric compound. In the work of [151], B_3_Si was reported as B_4_Si Olesinski and Abbaschian [143] suggested that different formulae were due to difficulty in phase separation of B_3_Si from B_6_Si, which shows a molar ratio of B to Si of around 4. However, X-ray analysis [45,152] revealed the composition of this compound as B_3_Si. Hence, in the current work, this compound is considered as B_3_Si. The B_n_Si compound was given the formula B_14_Si after Dirkx and Spear [153], who provided thermodynamic formation data for the B-Si intermetallic compounds. According to these authors [153], the intermediate phases were treated as line compounds and their formation enthalpies and entropies were assumed to be independent of temperature, claiming that it is a good approach when compounds exhibit narrow homogeneity ranges, although B_n_Si seems to have significant homogeneity range.

**Figure 10:**
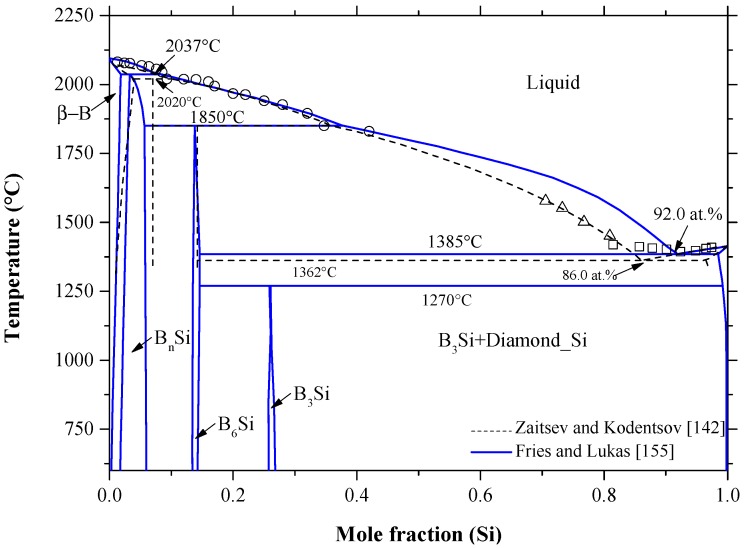
The B-Si phase diagram redrawn after [155]; ----: [142]; □: [144]; ○: [147]; Δ: [142].

Zaitsev and Kodentsov [142] modeled the B-Si phase diagram, based on their own thermodynamic measurements, shown as dashed lines in Figure 10. The modeled phase diagram [142] exhibits reasonable agreement with the assessment of Olesinski and Abbaschian [143] in the B-rich side up to 63.0 at % B. However, differences in other parts of the B-Si phase diagram could be observed. For instance, Zaitsev and Kodentsov [142] lowered the eutectic temperature from 1385 to 1362 °C with a shift in the eutectic composition from 86 to 92 at % Si, and therefore the liquid curve was suppressed in the 40–86 at % Si composition range. Moreover, Zaitsev and Kodentsov [142] treated B_n_Si as a stoichiometric compound, whereas Olesinski and Abbaschian [143] presented the compound with an appreciable homogeneity range. A comparison between the two phase diagrams [142,143] can be found in [154]. In this work, however, Figure 10 compares both recent calculated phase diagram versions by [155] and [142] with respect to the available experimental data [142,144,147]. 

**Figure 11:**
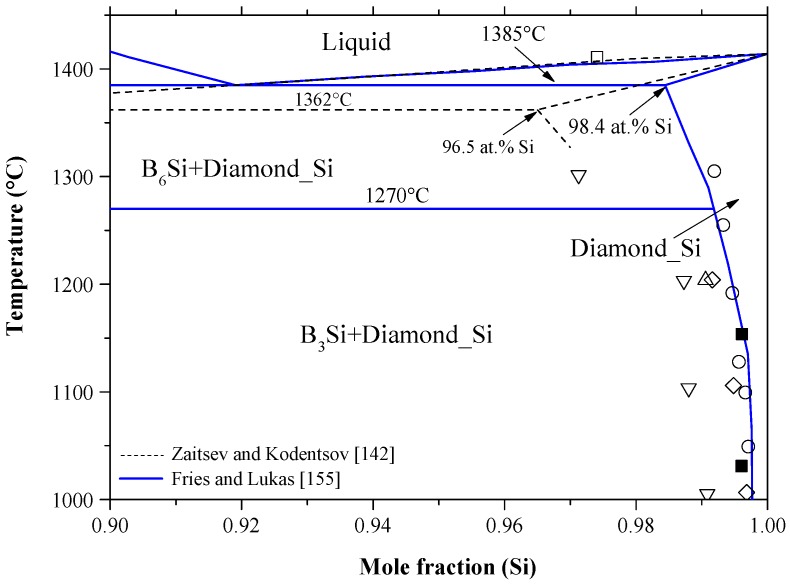
Magnified part of the B-Si system near the Si-rich side [155]; ----: [142]; Δ: [118]; ■: [157]; □: [144]; ▽: [145]; ◇: [146]; ○: [148].

The solid lines presented in Figure 10 are for the B-Si phase diagram calculated by Fries and Lukas [155] using the thermodynamic parameters presented in Table 7. These parameters are available in the FTlite database of FactSage^®^ software [124] and generate similar B-Si phase diagram to that of [155]. Equally, Seifert and Aldinger [156] performed a comprehensive review on the experimental and thermodynamic data of the B-Si system and presented a similar phase diagram. The calculated phase diagram [155] is in good agreement with the liquidus experimental results of [147] in the B-rich side as shown in Figure 10 and with those of the solvus reportedby [146,148,157] in the Si-rich side shown in Figure 11. However, the calculated liquidus line in the 70–92 at % Si composition range does not agree with the experimental points of Zaitsev and Kodentsov [142] and Brosset and Magnusson [144]. According to the limiting slopes of phase boundaries theory [139], the Si-rich liquid phase cannot be as flat as given by the experimental points of Brosset and Magnusson [144] and the calculations of Zaitsev and Kodentsov [142], because the liquidus is expected to be pushed upward by the peritectic formation of B_6_Si at 1850 °C. Thus, the liquidus at the Si-rich side must show steeper slope due to the relatively low eutectic point at 92 at % Si and 1348 °C. The thermodynamic calculations of the B-Si phase diagram, given by [155], are accepted in this work, because they better describe the phase diagram along with other thermodynamic properties of the solution phases and available experimental results.

The B solubility in Si data is scattered and contradictory. The solid solubility of boron in silicon as a function of temperature was first presented by Vick and Whittle [153]. According to these authors, the solid solubility varies from 9.0 × 10^−4^ at % at 700 °C to 1.4 × 10^−2^ at % at 1151 °C. Olesinski and Abbaschian [139], using different sources [114,141,142], estimated the B solubility in Si to be ~3.0 at %. Whereas, Zaitsev and Kodentsov [138] reported maximum solubility of 3.5 at % B at 1362 °C, favoring the results of Samsonov and Sleptsov [141] only. Although, the calculated solubility of B in Si by Fries and Lukas [151] is in good agreement with the data of [144,153] at 1.6 at %, further investigation of this region is highly recommended. Since the results of Armas et al. [143] and Vick and Whittle [152] support one another, a solvus line consistent with them is adopted in this work.

Boron is a fast diffuser in silicon. The intrinsic diffusivity of boron in silicon as function of temperature was represented by the equation $D_{i}=0.76\times e^{\left(-3.46\;eV/kT\right)}\;{\mathrm{cm}}^{2}/s$ [26]. Van-Hung et al. [158] calculated the diffusion coefficients of boron in silicon based on interstitial and vacancy mechanisms using statistical moment method. The temperature dependence activation energy and diffusion coefficients results [158] were compared with the available [26,159,160,161,162] literature data. They concluded that the dominant diffusion mechanism of B in Si is the interstitial mechanism [26,159,163]. It is worth mentioning that boron atom has 0.75 relative mismatch ratio with respect to, which is relatively large enough to produce high strain in silicon lattice. The induced lattice strain can reduce the level of other unwanted impurities by a trapping mechanism [26]. For example, high concentrations of boron are particularly good to combat iron impurities in Si [164].

**Figure 12:**
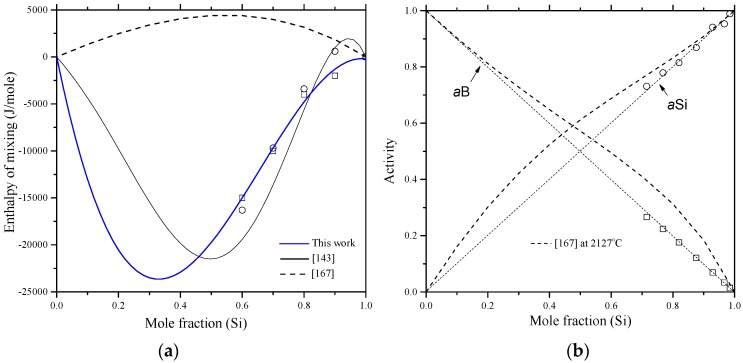
(**a**) Enthalpy of mixing of the B-Si liquid; —: [143]; □: [166,168] and ○: [150,165] at 1600 °C; -----: [167] at 2127 °C; (**b**) Average activity values of B and Si in the B-Si liquid; boron (□) and silicon (○) [142] at 1577 °C; ----: [167] at 2127 °C.

The enthalpy of mixing of B-Si melts, presented by a solid line in Figure 12a, was calculated by Olesinski and Abbaschian [143], who tried to fit their calculations with the experimental points given by Esin et al. [150]. Another set of experimental points were presented by Beletskii et al. [165], whose results agreed well with that of Esin et al. [150]. Later, Kudin et al. [166] determined the mixing enthalpies of B-Si melts in the 0.6 < X_Si_ < 1.0 composition range at 1600 °C using isoperibolic calorimetry. The enthalpy of mixing experimental data [166] were different from that of [143,150,165]. This could be attributed to the low purity of amorphous boron used by [166], which was about 98%. However, it is not logical to see a gradual increase and then decrease in the enthalpy of mixing curve [150,165] at the Si-rich side. Such behavior may result from the inaccurate measurements of the interaction energies in boron binary melts [166]. The recent thermodynamic assessments of the B-Si system [167] showed a positive enthalpy of mixing at 2127 °C as presented by a dashed line in Figure 12a. It seems that the liquid phase was treated as a random mixture, because the calculated enthalpy of mixing (4.4 kJ/mole) [167] was relatively small, which is not an accurate representation of the mixing in the B-Si melts. In this work, the enthalpy of mixing experimental results of Kudin et al. [166] are accepted for the B-Si system.

The activities of boron and silicon in the liquid phase were measured by Zaitsev and Kodentsov [142] using Knudsen effusion mass spectrometry in the 1249–1607 °C temperature range. The average activities of B-Si melts established by Zaitsev and Kodentsov [142] at 1577 °C show small deviation from Raoult’s low (negative for B and positive for Si), as shown in Figure 12b. The amount of deviation was found to increase with a decrease in temperature and increase in B concentration. It is important to note that the boron activity data points must obey Henry’s law in the Si side and not as reported by Zaitsev and Kodentsov [142]. Thus, we recommend further experimental efforts to measure the activity of B in the liquid phase. Due to the lack of reliable activity data, the boron and silicon activities at 2127 °C, reported by Franke and Neuschütz [167], are presented as dashed lines in Figure 12b. Both boron and silicon activities [167] show positive deviation from ideality, indicating that the solution is endothermic, which is contradictory to the enthalpy of mixing measurements of [166]. 

**Figure 13:**
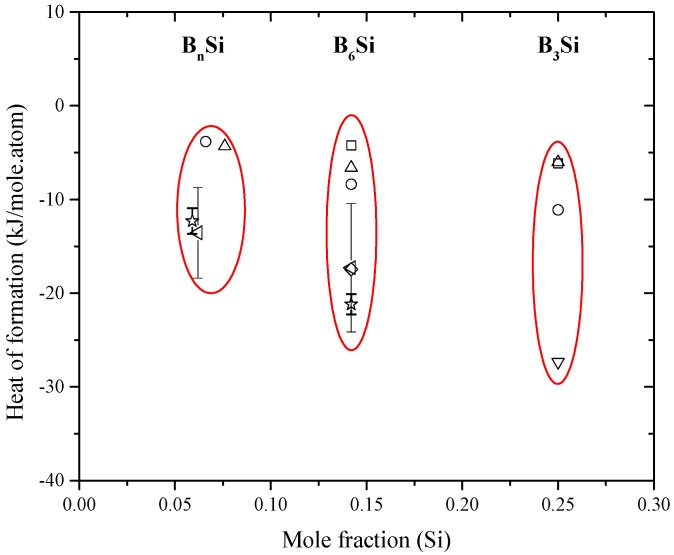
Enthalpy of formation of intermetallic compounds of the B-Si phase diagram; □: [155]; ○: [153]; Δ: [169]; ▽: [170]; ◇: [171]; ✰: [142]; ◁: [148].

According to data assessment in the current study, the B-Si phase diagram presented by Fries and Lukas [155] is accepted, since it matches with the phase diagram experimental and thermodynamic data. However, the calculated phase diagram [155] showed the maximum solubility of B in Si as 1.6 at % B at 1385 °C (as opposed to 3.5 at % at 1362 °C [142]), trying to fit their calculations with the experimental points of [146,148]. The enthalpies and entropies of formation of the intermetallic compounds values from different authors are scattered as could be seen in Table 8 and Figure 13.

### 5.4. The Bi-Si System

**Figure 14:**
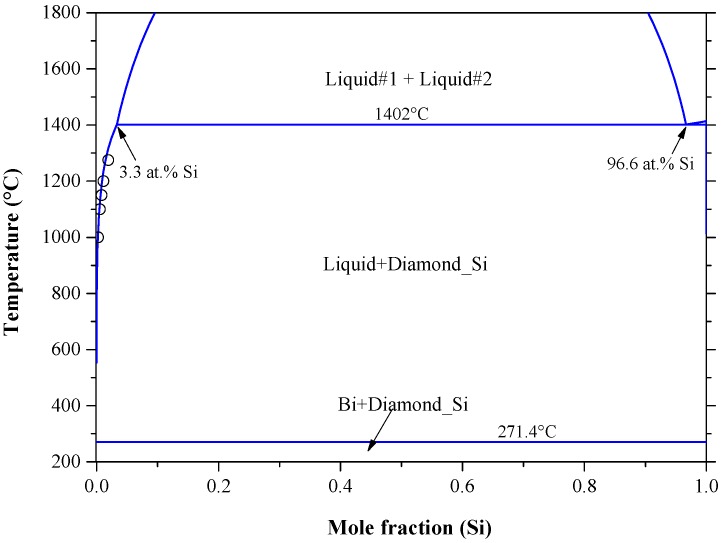
The calculated Bi-Si phase diagram [124]; ○: [120].

**Figure 15:**
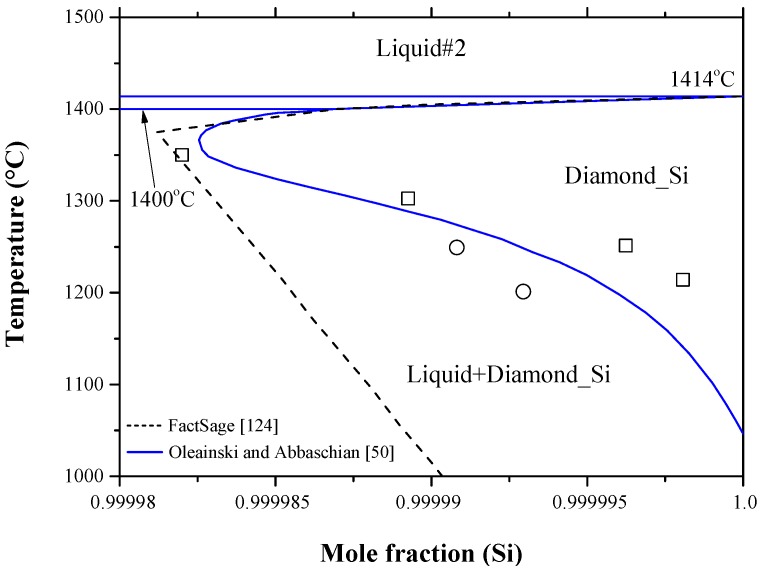
Magnified part of the Bi-Si system in the Si-rich side [50]; ----: [124]; ○: [179]; □: [118].

Bismuth atom is one of the shallow donor impurities in silicon. Bismuth-doped-silicon (Si:Bi) gained increasing interest due to its superior properties over the phosphorus/arsenic-doped-silicon (Si:P/Si:As) junctions [172,173,174,175]. The importance of bismuth-doped-silicon stems from its technological application as an infrared photoconducting detector [176]. The Bi-Si phase diagram was constructed by Olesinski and Abbaschian [50] based on the available experimental results and thermodynamic calculations in the literature [120,177,178,179]. Figure 14 shows the calculated Bi-Si phase diagram obtained from FTlite database and FactSage^®^ software [124], which is consistent with that of [50]. The system exhibits a monotectic transformation at 96.6 at % Si and 1402 °C; as well as a eutectic transformation at 1.7 × 10^−7^ at % Si and about 271.4 °C [66]. The solid solubility of Bi in Si showed a retrograde behavior and its maximum was estimated to be around 1.8 × 10^−3^ at % Bi at 1350 °C. Although the calculated [124] and assessed [50] maximum solubility of Bi in terminal Si side are consistent, the solvus line described by Olesinski and Abbaschian [50] is more representative, because it matches better with the experimental data of [118,179] as demonstrated in Figure 15.

The terminal solid solubility of Si in Bi is considered negligible [50,124]. The crystallographic data of the end-members of the Bi-Si system are given in Table 2.

**Figure 16:**
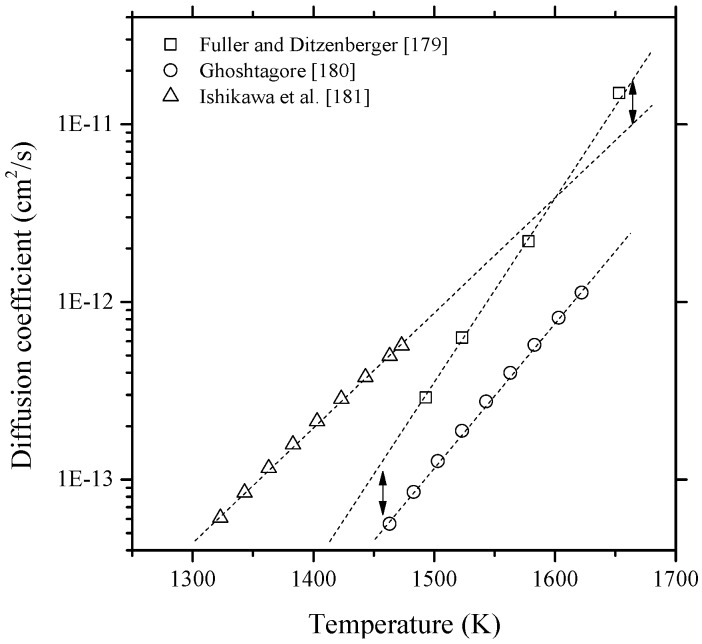
The temperature dependence of the diffusion coefficients, *D_Bi_*, in silicon; □: [179]; ○: [180]; Δ: [181].

Diffusion of Bi in silicon was investigated by several authors [179,180,181]. Fuller and Ditzenberger [179] expressed the diffusivity of bismuth in the 1220–1380 °C temperature range as $D=1030\times e^{\left(-1.109\;eV/\;kT\right)}\;{\mathrm{cm}}^{2}/s$ with an estimated error of about ±40%. This large error value was attributed to the concentration measurements at the junction depth due to the possibility of a Kirkendall effect [182], which would shift the reference diffusion interface. Ghoshtagore [180] concluded that bismuth diffusion in silicon obeys the point-defect mechanism. He measured the diffusivity of Bi in the 1190–1394 °C temperature range and expressed the diffusion equation as $D=1.08\times e^{\left(-3.85\pm 0.06\;eV/\;kT\right)\;}{\mathrm{cm}}^{2}/s$. Ishikawa et al. [181] could describe the diffusivity of Bi in Si, in the 1050–1200 °C temperature range, as $D=2.0\times 10^{-4}\times e^{\left(-2.5\;eV/\;kT\right)}{\;\mathrm{cm}}^{2}/s$. The temperature dependent diffusion coefficient of Bi in Si from different sources are plotted in Figure 16.

The measured diffusion coefficients were found to vary within one order of magnitude in the overlapped temperature range. It is interesting to note that if these measurements are extrapolated, measurements of [179] appear to be consistent with [181] at high temperature (~1650 K) and with [180] at low temperature (~1460 K). The slopes of the three curves, representing the activation energy of bismuth, show reasonable agreement with each other. However, the activation energy of bismuth was found much smaller than that of P, As and B, although they show similar diffusion coefficient values. This was attributed to the Bi atom-vacancy pairing mechanism [181].

**Figure 17:**
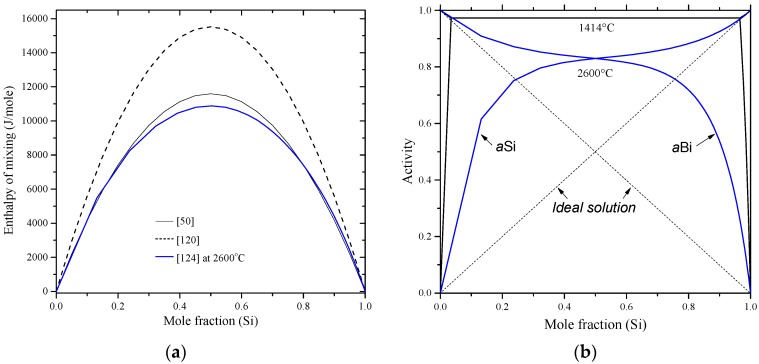
(**a**) Calculated enthalpy of mixing of the Bi-Si liquid [124] at 2600 °C; ―: [50]; ----: [120] and (**b**) calculated activity of Bi and Si in the Bi-Si liquid at 1414 °C and 2600 °C [124].

Recently, the Bi-Si binary phase diagram was calculated by Kaban et al. [183] using the parameters of liquid phase from the work of Olesinski and Abbaschian [50] given in Table 9. The enthalpy of mixing and activity of liquid silicon and bismuth are presented in Figure 17a,b, respectively. In Figure 17a, three enthalpy of mixing curves [50,120,124] are presented. Olesinski and Abbaschian [50] accepted the thermodynamic parameters of Thurmond [120] in their calculations. Differences in the enthalpy of mixing values are due to the use of different thermodynamic parameters for the liquid phase by [50,120] as shown in Table 9. Nevertheless, the calculated enthalpy of mixing of the Bi-Si liquid [50] is in good agreement with that of [124] at 2600 °C. The Bi and Si activities in the liquid phase are presented in Figure 17b based on the work of [67] at 1414 °C, which are identical to that of [124] at the same temperature. Additionally, the Bi and Si activities in the liquid phase were calculated at 2600 °C [124] to verify the findings in Figure 17a. The positive enthalpy implies that the mixing is endothermic and the attractive interaction between molecules of different species are weaker than those between molecules of the same species. This strong interaction between the pairs of similar atoms is reflective of the presence of immiscibility in the liquid phase. The shape of activity plot indicates the presence of a two-phase field at 1414 °C, which is liquid #1 and liquid #2 as inferred from the Bi-Si phase diagram in Figure 14.

### 5.5. The Ga-Si System

**Figure 18:**
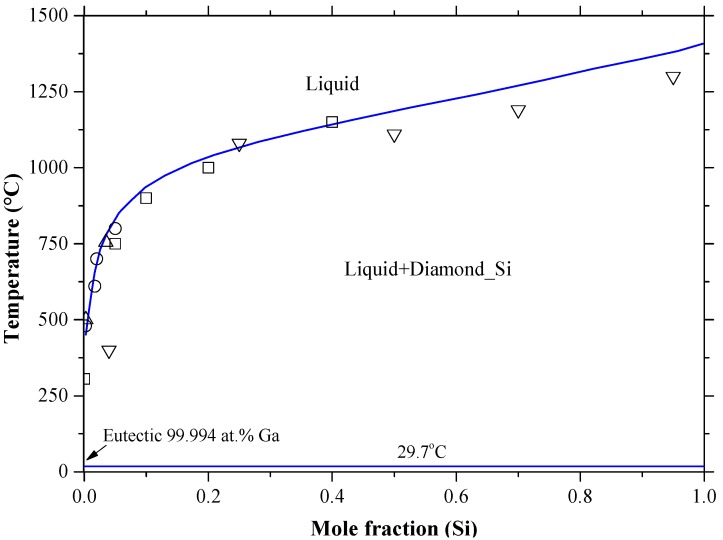
The Ga-Si phase diagram [185]; □: [186]; Δ: [188]; ▽: [187]: ○: [178].

**Figure 19:**
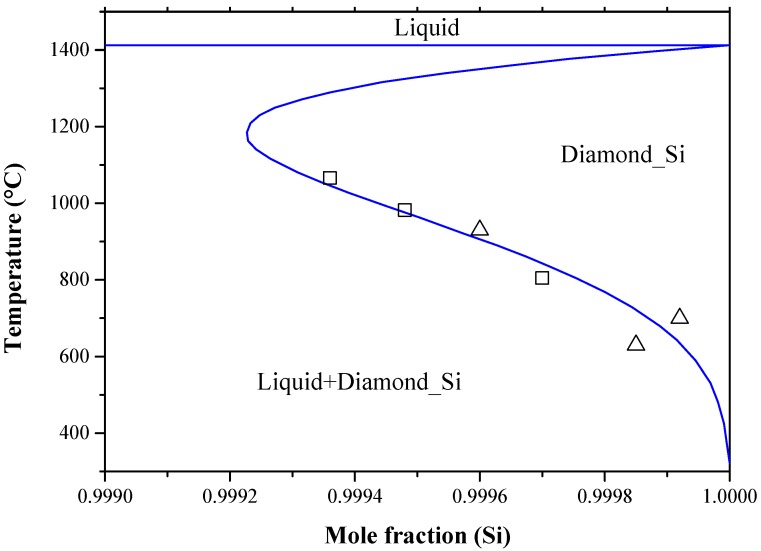
Magnified part of the calculated Ga-Si phase diagram [185]; □: [118]; Δ: [85].

Gallium, as a *p*-type dopant in silicon, is attaining great interest for the PV industry, because silicon thin films can be grown in Ga melts by liquid phase epitaxy [85,184]. The Ga-Si binary phase diagram, shown in Figure 18, was redrawn after Franke and Neuscütz [185], who accepted the phase diagram data reported by Olesinski et al. [51]. The system is described as a simple eutectic that occurs at 99.994 at % Ga and 29.7 °C [185]. The solid solubility of gallium in silicon shows typical retrograde behavior, as demonstrated in Figure 19, and was estimated as ~0.08 at % at around 1200 °C [51]. The solubility of silicon in gallium is negligible, because the liquid phase almost degenerate on the Ga terminal side. The crystallographic data of the end members of the Ga-Si system are given in Table 2. The liquid phase was experimentally investigated through thermal analysis [186], and thermal analysis and metallography [187]. The liquid solubilities were determined by a weighing technique in the 300–800 °C temperature range [178,188]. The liquidus of Ga-Si phase diagram [185] shows a reasonable agreement with the experimental data of [178,186,188]. However, Savitskiy et al. [187] reported lower values due to impurities in the starting elements. It is worth mentioning that the retrograde solubility of Ga in Si is an indication of a large entropy of the Si-Ga solid solution with temperature [189].

Gallium was used at the beginning of the semiconductor industry for making alloy junctions, because of its high diffusion coefficient and low eutectic temperature [90]. However, the use of gallium is limited, because of its high diffusivity in SiO_2_, which is commonly used for layer masking [190]. The diffusion of gallium in silicon was first investigated by Fuller and Ditzenberger [179] who described the diffusion coefficient of gallium in the 1105–1360 °C temperature range as $D=3.6\times e^{\left(-3.5eV/kT\right)}\;{\mathrm{cm}}^{2}/s$. In agreement with Fuller and Ditzenberger [179], Pichler [90] derived the diffusivity of gallium under intrinsic and inert conditions in silicon from several studies [179,191,192,193,194] as $D_{i}=3.81\times e^{\left(-3.552\;eV/kT\right)}\;{\mathrm{cm}}^{2}/s$, in the 800–1380 °C temperature range.

**Figure 20:**
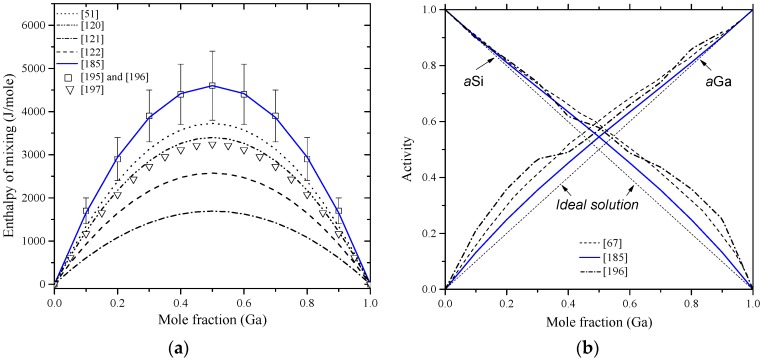
(**a**) Enthalpy of mixing of the Ga-Si liquid at 1477 °C; **―**: [185]; □: [195] and [196] at 1487 ± 5 °C; **-**·**-**·**-** : [121]; **-**··**-**··**-**: [120]; -----: [122]; ▽: [197]; ···· : [51] at >1414 °C and (**b**) Activity of gallium and silicon in the Ga-Si liquid; **―**: [185]; -·-·-: [196] and ------ : [67] at 1477 °C.

Enthalpy of mixing of the liquid Ga-Si binary alloys were measured at 1487 ± 5 °C by Kanibolotsky et al. [195], using high-temperature isoperibolic calorimetry, up to 0.6% Ga. The enthalpy of mixing for the melt of these alloys was found positive, as shown in Figure 20a, which is an indication of an endothermic mixing. In a recent investigation, Sudavtsova et al. [196] reported mixing enthalpies of the Ga-Si melt in the full composition range, using isoperibolic calorimetry at 1477 °C, and their results were in good agreement with those of Kanibolotsky et al. [195]. Thus, the enthalpies of mixing determined by [195,196] using calorimetric measurements are recommended in this work as they are more reliable. The activities of gallium and silicon in the melts at 1477 °C are given in Figure 20b. Sudavtsova et al. [196] calculated the Si activity from the coordination of the liquidus line using Schröder’s equation, while the Ga activity was calculated using the analytical-numerical Gibbs-Duhem integration. The calculated activities of Ga-Si melts by Safarian et al. [67] were based on the work of Sudavtsova et al. [196]. The recently assessed activities of Ga and Si in the melts [185] deviate from the calculations of [67,196] and show nearly ideal behavior. This deviation could be due to that Sudavtsova et al. [196] treated the Ga-Si melt as quasi-regular solutions. Moreover, they correlated the positive deviation from ideality to the absence of compounds and/or solid solutions in the system and to the degenerate eutectic in the Ga-rich side. The optimized model parameters for the liquid phase, listed in Table 10, were adopted by Franke and Neuscütz [185] based on the work of Olesinski et al. [51].

### 5.6. The In-Si System

**Figure 21:**
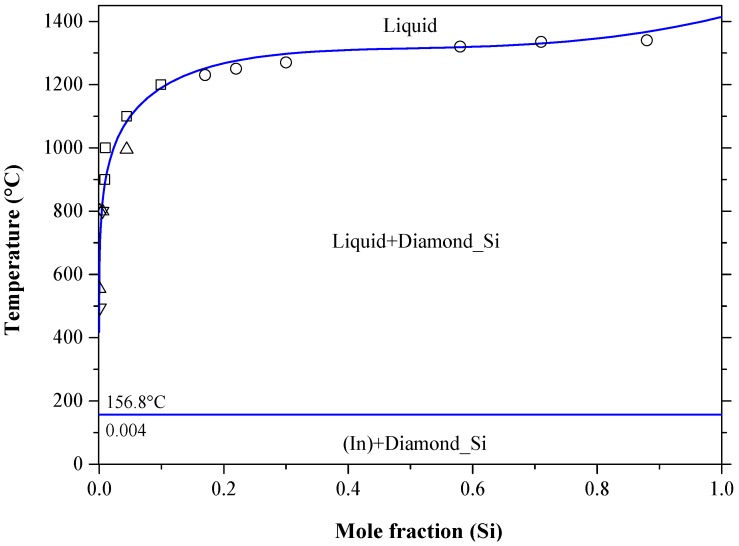
The calculated In-Si phase diagram **―**: [124] based on the assessment of [52]; ○: [186]; □: [120]; Δ: [188]; ▽: [178].

Indium is a *p*-type impurity in silicon from group IIIA of the periodic table. Indium has been used for the fabrication of infrared detectors, because of its high ionization energy [90]. The binary In-Si phase diagram was assessed by Olesinski et al. [52] using the experimental data of the liquid phase from [120,178,186,188] and the solubility data of In in Si from [198,199]. The In-Si binary phase diagram shown in Figure 21 was calculated using FTlite thermodynamic database [124]. The calculated phase diagram [124] is consistent with the assessment of Olesinski et al. [52]. The system shows a flat liquidus, which indicates the existence of a metastable miscibility gap in the liquid. A eutectic transformation, occurs at 4 × 10^−3^ at % Si and very close to the melting temperature of In. The crystallographic data of the end members of the In-Si system are listed in Table 2. The intrinsic diffusivity of indium in silicon as function of temperature, from experimental measurements of [179,200,201,202], was derived by Pichler [90] to be as follows: $D_{i}=3.13\times e^{\left(-3.668\;eV/kT\right)}{\;\mathrm{cm}}^{2}/s$.

**Figure 22:**
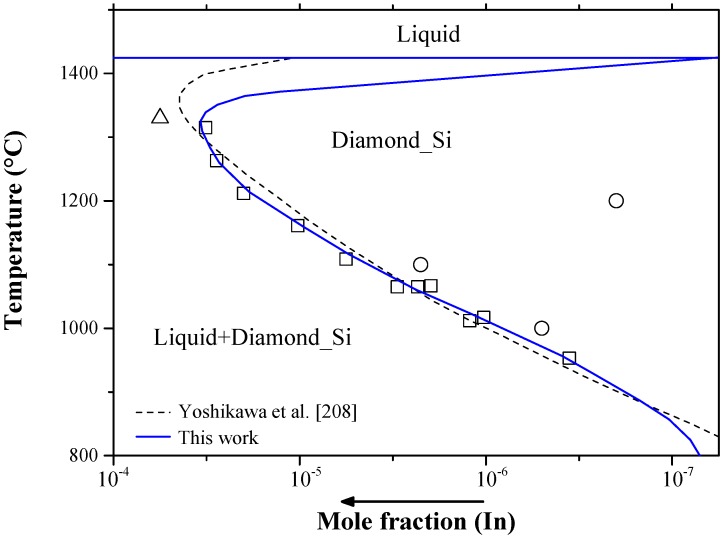
Magnified part of the In-Si phase diagram; -----: [208]; □: [204]; ○: [200]; Δ: [198].

Different maximum solid solubility values of indium in silicon were reported by Backenstoss [198] as 8 × 10^−4^ at % using neutron activation analysis and by Jones et al. [199] as 5 × 10^−3^ at % at 1330 °C using a gradient transport solution growth process. Pichler [90] provided values for the solid solubility of indium in silicon from resistivity combined with Hall-effect measurements [200,201,202,203,204,205,206] or capacitance-voltage profiling [207] at different concentrations and temperatures. Later, the solid solubility of In in Si was calculated by Yoshikawa et al. [208], considering the results from the work of Scott and Hager [204], as shown in Figure 22. In their calculations [208], the liquidus was presented as a dashed line due to the lack of experimental data [204] at high temperatures. The maximum values of indium solubility in silicon, reported by Scott and Hager [204], was only up to 1300 °C, because of the furnace limitations and problems in quartz ampoules. This explains why the shape of In solubility curve did not decrease to zero at the Si melting temperature (i.e., retrograde solubility) as typically observed in other systems, such as Al-Si, As-Si, Ga-Si and Sb-Si. Later, Cerofolini et al. [200], using resistivity and Hall measurements, reported lower indium solubility in silicon due to the high diffusivity of In atoms in Si lattice, which allows the existence of a metastable state, observed by Cerofolini et al. [209], in the In-Si system. The solubility limits of indium in silicon [200] were measured using ion implantation followed by thermal annealing. Although this technique is operated at low temperatures, which means that doping can be performed without influencing previously diffused regions and produce uniform In-doped layers, the solvus line is considered inaccurate. This conclusion was drawn from Figure 22. The experimental data point at 1200 °C [200] indicate that the solubility of In did not start at the Si melting point. On the other hand, Scott and Hager [204] prepared indium-doped-silicon using a gradient-transport solution growth process in the 950 to 1300 °C temperature range and measured the In solubility using Hall measurements. Although this technique promotes structural defects with increasing temperature, the solubility measurements of indium in silicon proves to be accurate for the whole temperature range as can be seen in Figure 22. In agreement with [204], the accepted Si-rich solvus line in the current study is presented by a solid line in Figure 22.

**Figure 23:**
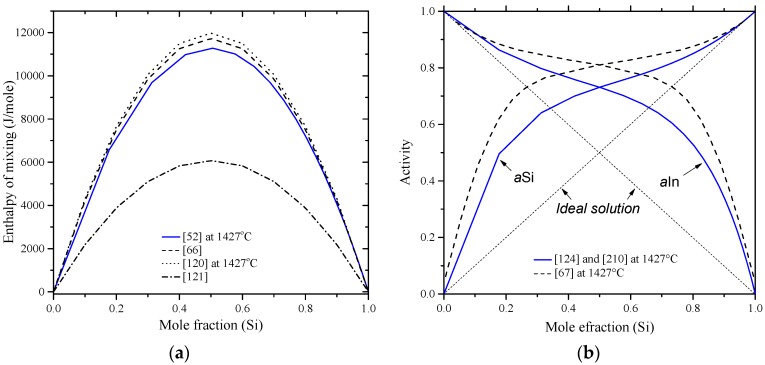
(**a**) Enthalpy of mixing of In-Si melts at 1427 °C [52]; -·-·-: [121]; ····: [120]; -----: [66] at 1427 °C and (**b**) Activity of In and Si in the liquid In-Si phase [124,210]; -----: [67] at 1427 °C.

The thermodynamic properties of the In-Si system were reviewed by Frank and Neuschütz [210] based on the work of Olesinski et al. [52] and Tmar et al. [197]. Enthalpy of mixing and activity are given in Figure 23a,b, respectively. The enthalpy of mixing values of In-Si melts found in the literature [52,66,120] support one another and thus are accepted in the current work. The results of Thurmond [121] were not considered in the current evaluation, because they show different enthalpy of mixing values of about 6000 J/mole at mid-composition from other results [52,66,120]. The Si-In liquid solution is endothermic as indicated by the positive enthalpy of mixing and positive deviation of activities from Raoult’s law. The optimized model parameters for the liquid phase [52] are listed in Table 11, which represent the most acceptable data for the In-Si phase diagram.

### 5.7. The N-Si System

**Figure 24:**
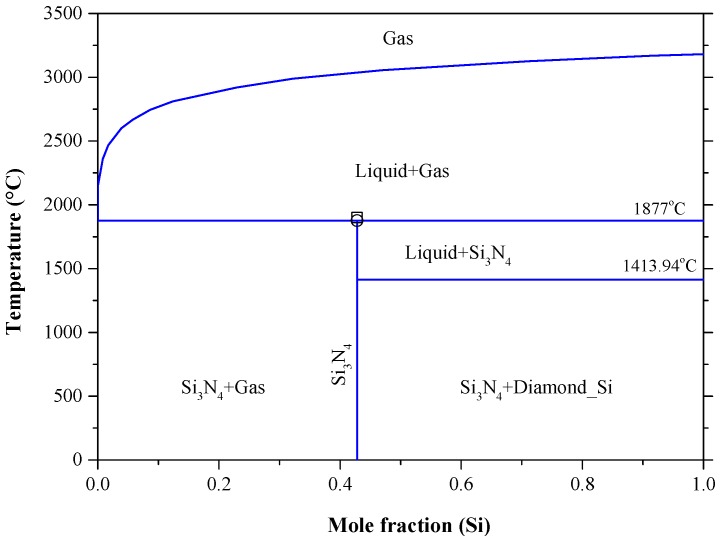
The N-Si binary phase diagram calculated by Ma et al. [214] at 1 atm; ○: [221]; □: [223].

**Figure 25:**
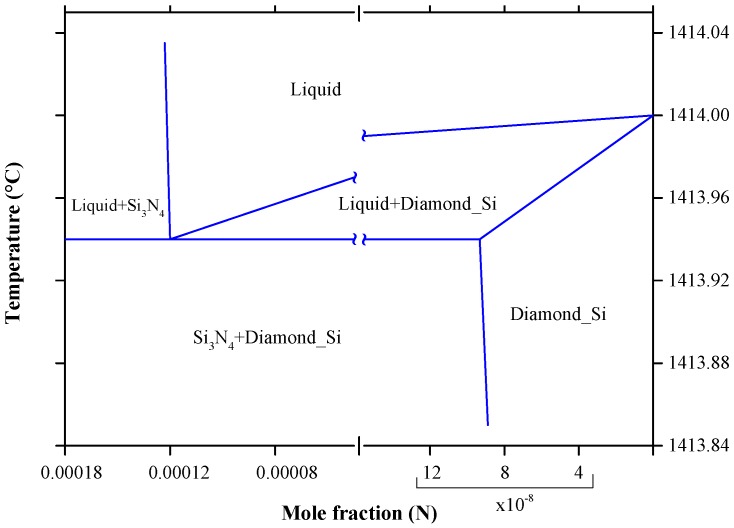
The N-Si partial phase diagram for the extremely low N concentration after Yatsurugi et al. [32].

Nitrogen gas is often used as inert atmosphere during post implantation annealing and as a carrier gas in semiconductor diffusion technology [211]. Therefore, the commercial silicon may contain nitrogen in dissolved form if no measures for cleaning are taken [32]. Kaiser and Thurmond [212] found that nitrogen in silicon have donor properties when it reacts with liquid silicon and forms silicon nitride (Si_3_N_4_). The thin film silicon nitrides are attractive for applications in microelectronic and optoelectronic devices, because they perform as gate dielectric layers, intermetal insulators, passivation films, diffusion barriers or optical matching layers [213,214]. The N-Si phase diagram, in Figure 24, was developed by Ma et al. [214], using Calphad method at 1 atmosphere, taking into consideration the available data [215,216,217,218,219,220,221,222]. At the same pressure, the system exhibits a eutectic reaction of L → Diamond Si + Si_3_N_4_ at around 1413.94 °C and 0.012 at % N as demonstrated in the N-Si partial phase diagram for extremely low N concentrations redrawn after Yatsurugi et al. [32] in Figure 25. Using zone-melting experiments, the maximum solubility of N in Si was found to be 9 × 10^−6^ at % at the eutectic temperature [32]. The extremely low solubility of N in Si was attributed to the high stability of Si_3_N_4_ besides the large difference in the atomic radii of N and Si [32]. At the same eutectic temperature, Kaiser and Thurmond [212] reported a much lower solubility of N in solid Si as 2 × 10^−9^ at % and a higher N concentration of 0.02 at % in liquid Si due to the detectability limit of the measurements.

Despite their low solubility, nitrogen impurities strongly interact with vacancies in Si lattice altering the size and kinetics of the voids causing gate oxide failure [224]. Using dislocation unlocking experiments, Alpass et al. [225] expressed the effective diffusivity of nitrogen in Si in the 500–750 °C temperature range as: $D_{eff}=200\;000\times e^{\left(-3.24\;ev/kT\right)}\;{\mathrm{cm}}^{2}/s$. According to Fujita et al. [224], the di-interstitial nitrogen defect was found to be the dominant diffusion mechanism of nitrogen in Si. In this mechanism, two nitrogen interstitial atoms bind into Si lattice due to their rapid diffusion. The defects of this type are known to be stable up to 900 °C [224]. Nevertheless, they do not possess any deep donor or acceptor properties [226].

**Figure 26:**
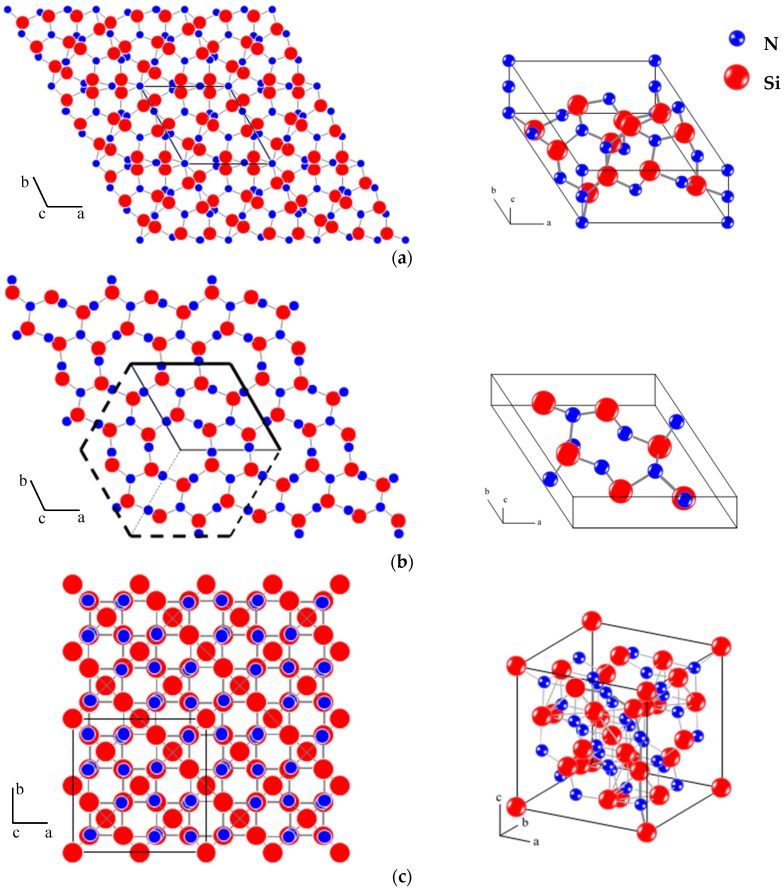
Crystal structure and unit cell of (**a**) αSi_3_N_4_, (**b**) βSi_3_N_4_, showing the hexagonal symmetry, and (**c**) γSi_3_N_4_.

So far, the Si_3_N_4_ [227] is the only confirmed intermetallic compound in the N-Si system [228]. The presence of other silicon nitrides, such as SiN [229,230], Si_3_N_2_ [230], Si_3_N [231] and Si(N_3_)_4_ [232], was uncertain and not supported by other studies. The compound Si_3_N_4_ exists in three polymorphic forms, α, β and γ phases [233]. Analogous to cubic boron nitride, cubic γSi_3_N_4_ with a spinel-type structure exists but only at high pressure (above 15 GPa) and temperature (exceeding 2000 K) [234]. Therefore, it will only be mentioned briefly here and more importance will be given to α and β structures. According to Grün [54], βSi_3_N_4_ is the more stable phase at room temperature and αSi_3_N_4_ is a metastable phase. The crystallographic data of Si_3_N_4_ phases are presented in Table 2 and Table 12. In principle, both α and β silicon nitride phases exist as stoichiometric compositions with Si_12_N_16_ for α-phase and Si_6_N_8_ for β-phase [235], representing the 28 and 14 atoms in the unit cells, respectively. Whilst αSi_3_N_4_ has trigonal symmetry with space group *P3*_1_*c* (No. 159) [235] and Pearson symbol hP28 [39], βSi_3_N_4_ has hexagonal symmetry with space group *P6*_3_ (No. 173) [235] and Pearson symbol hP14 [39]. Figure 26 is drawn to describe the crystal structure of the three forms of Si_3_N_4_ based on the crystallographic data from Table 2 and site occupancies from [39] in Table 12. It can be depicted from the crystallographic data and the graphic presentation of silicon nitride phases that Si_3_N_4_ tetrahedra in the α structure appear as interconnected cavities as opposed to tunnels in β structure parallel to the *c*-axis of the unit cell. The length of *c* parameter in αSi_3_N_4_ is almost double that of βSi_3_N_4_. This is attributed to the occupancy of additional Si2 atoms on 6c sites of the unit cell.

**Figure 27:**
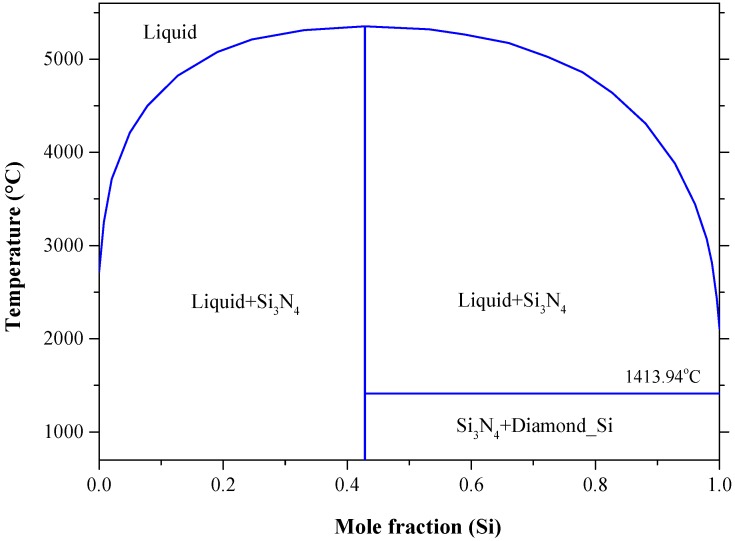
The calculated condensed N-Si phase diagram at 7.5 × 10^9^ Pa [214].

The N-Si phase diagram in Figure 24 shows that Si_3_N_4_ has no true melting temperature at atmospheric pressure. It rather decomposes to liquid and N_2_ gas at 1877 °C [214,221], which is only 23 °C lower than the measurement reported by Forgeng and Decker [223] as 1900 °C. Although, in this work we consider the values reported by [214,221,223] are consistent, the variation between them could be due to the pressure difference between these measurements. For instance, Forgeng and Decker [223] did not specify the pressure in their work; while both [214,221] performed their calculations at the atmospheric pressure. Because of no other compounds in the N-Si system, it is expected that Si_3_N_4_ melts congruently at high enough nitrogen pressure as shown in the calculated condensed N-Si phase diagram [214] in Figure 27. The maximum calculated congruent melting temperature of Si_3_N_4_ is 5318 °C at about 7.5 × 10^9^ Pa.

Because of the very small solid solubility of N in Si , the Diamond Si terminal solid solution was treated as pure Si in the calculated N-Si phase diagram of Ma et al. [214]. However, the calculated composition of the L → Diamond Si + Si_3_N_4_ eutectic is in good agreement with that of Yatsurugi et al. [32] at 0.012 at % N. This consistency indicates that the eutectic composition is not affected by the pressure. Overall, the N-Si phase diagram optimized by Ma et al. [214] is acceptable in this work. The optimized model parameters and enthalpy and entropy of formation of Si_3_N_4_ are listed in Table 13 and Table 14, respectively. The Gibbs energy of the gas phase is taken from SGTE substance database [236] and that of the pure elements are from Dinsdale [237]. The enthalpies and entropies of formation for Si_3_N_4_ reported by Ma et al. [214] were mainly taken from the work of Pehlke and Elliott [216] and the heat capacity values were from Guzman et al. [217].

### 5.8. The P-Si System

Silicon-doped-phosphorus is very important for the power semiconductors [90] application. Phosphorus is the fastest diffusing donor impurity in silicon. However, the maximum required limit of phosphorus for solar-grade Si is almost 10^−5^ at %. At higher concentration, phosphorus introduces lattice strain due to the tetrahedral mismatch ratio between P and Si atoms of about 93%. The misfit lattice strain narrows the band gap, and reduces the (PV)^−^ pair dissociation [26]. This infinitesimally small amount makes phosphorus refining process during recycling very difficult, because phosphorus has a relatively large segregation coefficient, *K_o_*, in silicon of about 0.35 [238,239]. 

Diffusivity of phosphorus in silicon has been measured using several experimental and analytical techniques as reviewed by Christensen [240]. The intrinsic diffusivity of phosphorus in silicon was expressed, by Fair [137], as: *D_i_ = *3.85 *× e*^(−3.66 *eV/kT*)^ cm^2^/s. Later, Van-Hung et al. [158] calculated the diffusion coefficients of phosphorus in silicon by interstitial and vacancy mechanisms using statistical moment method. They concluded, by comparing their own results with the available data [26,159,163], that the dominant diffusion mechanism of P in Si is the interstitial mechanism, similar to boron.

**Figure 28:**
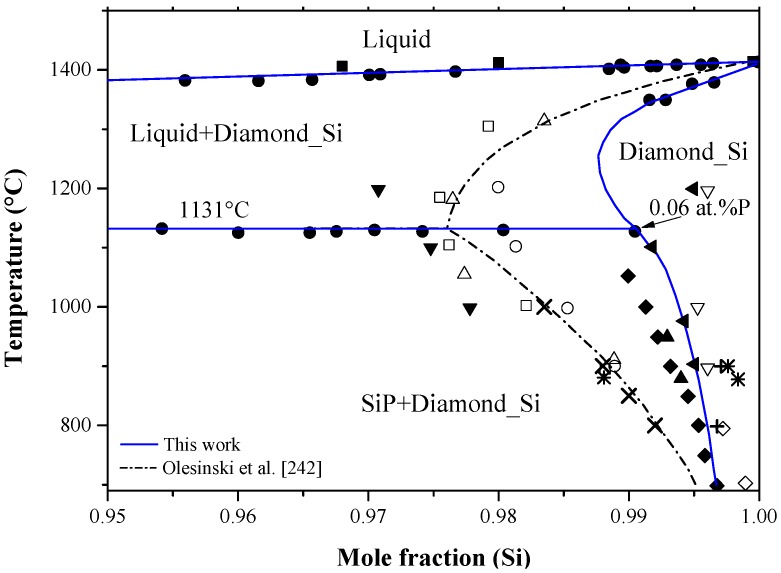
Assessed Si-terminal solid solution in relation to the available data; -·-·-·-: [242]; □: [118]; ■: [241]; Δ: [243]; ◇: [244]; ▽: [245]; **×**: [246]; +: [247]; ▲: [248]; *: [250]; ▼: [252]; ○: [249]; ♦: [253]; ◄: [254]; ●: [255].

The phase diagram and thermodynamic properties of the P-Si system are still under investigation, because of the difficult experimental requirements due to the presence of liquid-vapor phase boundaries [55]. First version of the P-Si binary phase diagram was revealed by Giessen and Vogel [241]. According to these authors, the eutectic temperature and the liquidus were determined using thermal analysis in the 0 to 28.0 at % P composition range. No or very limited solubility of P in the Si terminal side was assumed and a stoichiometric intermetallic compound of SiP was proposed based on the lever rule calculations [241]. Additionally, they attempted to measure the equilibrium gas composition by quenching alloys from liquid-vapor region using the knowledge of the starting composition, liquid composition and mass change in P-Si alloys. Olesinski et al. [242] reproduced the P-Si phase diagram at ambient pressure based on the liquidus and eutectic data from Giessen and Vogel [241] and the solubility of P in Si from Kooi [243] data, as their solubility limits [243] were found more convincing by [242]. Whereas, the solubility of Si in P is considered negligible [242]. The solubility of P in the Si terminal side was investigated by many authors [118,243,244,245,246,247,248,249,250,251,252,253,254,255] but the reported data show large discrepancies among each other due to sample oxidation, the presence of impurities, or other experimental difficulties. According to Trumbore [118], the solid solubility of phosphorus in silicon shows a retrograde behavior with a maximum value of about 2.50 at % P at 1180 °C, which is close to 2.4 at % obtained by Kooi [243], who studied the diffusion behavior of P in Si in the 920 to 1310 °C temperature range using a neutron activation analysis. Abrikosov et al. [244] used the microhardness measurements to determine the solubility of P in Si with a maximum of about 1.0 at % at 900 °C. On the other hand, lower concentrations were obtained by Uda and Kamoshida [245] and Tamura [249] at 1100 °C as about 0.3 at % and 0.6 at % P, respectively, upon measuring the P concentration in the ion-implanted and annealed Si crystals. Solmi et al. [246] used secondary neutral mass spectrometry measurements to determine the solubility of P in Si from 800 to 1000 °C. Nobili et al. [253] used Hall effect and resistivity measurement to determine the solubility of P in an ion-implanted Si specimens. The high values reported by [118,243] were not reliable as Nobili et al. [253] pointed out. The problem in Trumbore’s [118] data was due to using unjustified error function when the surface concentration was calculated. Moreover, the data given by Kooi [243] were related to O-P-Si ternary and not for P-Si system, due to the presence of an oxide film on the substrate. Although these data were criticized by Nobili et al. [253], they were used by Olesinski et al. [242] and Jung and Zhang [256] for P-Si system evaluation and optimization. Safarian and Tangstad [255] reinvestigated the liquidus and solidus of the Si-rich side of the P-Si phase diagram, up to 5.47 at % P, using thermogravimetric and differential thermal analysis (TG/DTA) experiments. According to these authors, the solubility of P in Si shows a retrograde behavior with 0.06 at % P at the eutectic temperature 1129 ± 2 °C and 0.09 distribution coefficient of P in Si. Furthermore, they [255] estimated the solubility line below eutectic based on the data of Borisenko and Yudin [254], because they [255] considered the high P solubility values as coexisting P in SiP precipitates. More recently, Liang and Schmid-Fetzer [257] in their optimized P-Si phase diagram classified the experimental solubility data into lower and higher solid solubilities near the eutectic temperature and thus used two different sets of thermodynamic parameters for the diamond-Si phase optimization. Figure 28 compares different sets of experimental and calculated data in the Si-rich side of the P-Si binary phase diagram.

Thus far, the available experimental and calculated solubility limits data of P in Si are scattered and difficult to use to draw a common diamond-Si phase boundary. However, the experimental points provided by Safarian and Tangstad [255] are more convincing for the following reasons. The purity of the starting materials was 99.9999 wt % Si and 99.999 wt % P. Furthermore, authors used precise characterization techniques, such as wavelength dispersive spectroscopy (WDS) over many points for each measurement to determine the composition of the alloys and TG-DTA to determine the liquidus and solidus phase boundaries in the Si-rich side. The DTA results gave logical linear liquidus and solidus relationships below 5.5 at % P if the limiting slope theory is considered. 

Only one intermetallic compound, SiP, exists in the P-Si system at ambient pressure as presented by Olesinski et al. [242]. However, Liang and Schmid-Fetzer [257] reported two intermetallic compounds, SiP_2_ and SiP, in their calculated phase diagram at 0.5, 1, and 200 bar. The compound Si_2_P was only reported by Fritz and Berkenhoff [258], using X-ray and infra-red (IR) spectra, to form at 450 °C and decompose into Si and SiP at 600 °C. Due to the limited information, the compound Si_2_P was not included in different versions [55,242,256,257] of the P-Si phase diagram. SiP single crystal was obtained by Beck and Stickler [40], who determined the structure of SiP as orthorhombic using X-ray diffraction (XRD) and transition electron microscopy (TEM). The crystal structure of SiP in Table 2 was reported based on Wadsten [259] investigations. The SiP_2_ phase was first reported by Wadsten [42], who investigated the phosphide alloys using XRD. Later, SpringThorpe [260] confirmed the existence of SiP_2_ by synthesizing a large crystal of orthorhombic structure using vapor transport technique [261]. The SiP_2_ crystal developed by SpringThorpe [260] was twice longer in b-direction (*b* = 2.006 nm) as that proposed by Wadsten [42] (*b* = 1.008 nm). It could be that the measurements of [42] and [260] were performed on a deformed crystal. In the current study, the accepted crystal structure of SiP_2_ was based on the work of Wadsten [42], since it was used and republished by [39]. A new silicon phosphide Si_12_P_5_, with rhombohedral symmetry and C_5_W_12_ structure, was reported by Carlsson et al. [262]. The phase Si_12_P_5_ was found to form in an amorphous P-Si alloy thin film after annealing at 1000 °C for 30 min and dissociates at above 1050 °C. Hence, only two intermetallic compounds, SiP and SiP_2_, are accepted in the current work for the equilibrium phase diagram of the P-Si system, because their formation, structure and properties have been well described and confirmed in the literature [40,42,259,260]. 

The vapor composition and thermodynamic properties of the P-Si melt, containing 0.09–26.5 at % P, were investigated by Zaitsev et al. [263] in the 1234–1558 °C temperature range. The chosen range covers almost the liquid phase field. The Gibbs energies of phase transitions of phosphorus and silicon were taken from [264,265]. The calculated phosphorus activities were in good agreement with their [263] own experiments. However, Zaitsev et al. [263] calculated the equilibrium of liquid, solid silicon and vapor phases at 1166 °C, which was higher than that reported by Giessen and Vogel [241] as 1131 °C. The variation could be due to the difficulty in differentiating between the eutectic and primary solidification in this system. Franke and Neuschütz [55] presented a calculated P-Si phase diagram based on the literature data [114,115,239,241,242,263]. Similar version was obtained using FTlite thermodynamic database in FactSage^®^ software [124]. According to [55], the terminal solid solution in the Si-rich side was represented as a typical solid solution, with maximum solubility at the eutectic line, and not as a retrograde solution. Jung and Zhang [256] reassessed the P-Si phase diagram using the modified quasi-chemical model [266] for the liquid phase and compound energy formalism [267] for Si-rich phase assuming P substitutes Si. According to these authors, the thermodynamic properties of SiP were re-optimized using low-temperature heat capacity of Ugai et al. [268] and the modified high-temperature heat capacity parameter of Knacke et al. [269]. Whereas, the thermodynamic properties of SiP_2_ were based on the heat capacity measurements of Philipp and Schmidt [270]. The evaporation and gas composition data [241] were not taken into account in the thermodynamic modeling of Jung and Zhang [256], because they [256] could not reproduce the experimental results.

**Figure 29:**
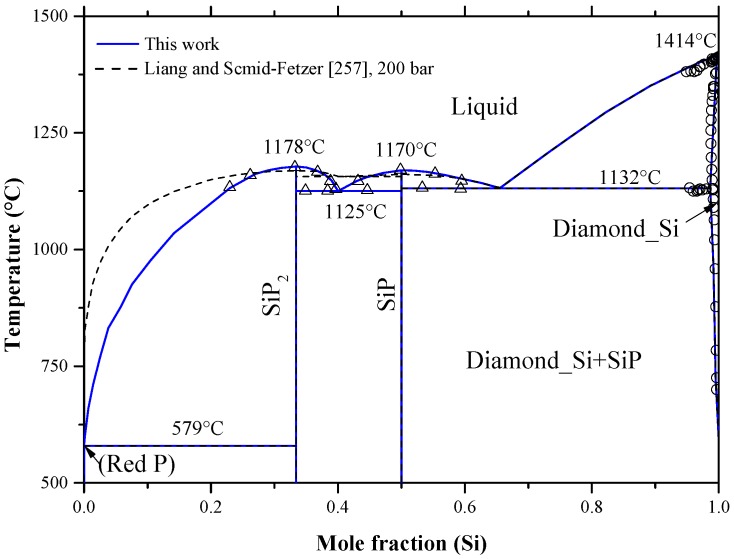
The P-Si phase diagram without gas phase at 200 bar; -----: [257]; Δ: [268]; ○: [255].

Recently, Liang and Schmid-Fetzer [257] remodeled the P-Si phase diagram considering no short-range-ordering in the liquid phase. In their work, the liquid phase and Si-rich terminal solution were described using the substitutional solution model, SiP and SiP_2_ were treated as stoichiometric compounds and the gas phase was described as an ideal gas mixture. Furthermore, new set of thermodynamic parameters were provided [257], because they discovered some errors in the published parameters by Jung and Zhang [256]. The optimized P-Si phase diagram at 200 bar [257] along with the experimental data [255,268] is shown in Figure 29. The optimized model parameters of the P-Si phase diagram are listed in Table 15.

In agreement with [256], the corresponding gas composition points [241] were not used by Liang and Schmid-Fetzer [257] in their optimization, because the data were considered inaccurate. At 200 bar, both SiP and SiP_2_ compounds melt congruently at 1161 °C and 1169 °C, respectively, which are slightly below the experimental data [268] as shown in Figure 29. In this work, modified phase boundaries are based on the experimental data of Ugai et al. [268], who measured the congruent melting of SiP and SiP_2_ to be 1170 °C and 1178 °C, respectively, and the SiP + SiP_2_ eutectic at 1125 °C. The enthalpy and entropy of formation of the P-Si compounds are given in Table 16. It is worth noting that the enthalpy and entropy of formation of SiP, used in the calculations of Liang and Schmid-Fetzer [257], were similar to those given by [256]. Whereas, the thermodynamic parameters of SiP_2_ were slightly modified by [257] to fit with the experimental congruent melting point as given by Ugai et al. [268].

**Figure 30:**
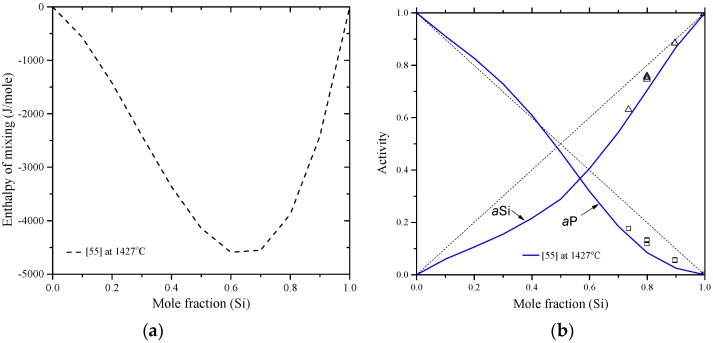
(**a**) Calculated enthalpy of mixing of the P-Si liquid at 1427 °C; ----: [55]; (**b**) activity of Si and P in the P-Si liquid at 1427 °C; ----: [55]; □ and Δ: [263].

Very limited experimental data on the activity of P-Si in the melts could be found in the literature. Zaitsev et al. [263] investigated the activity of the P-Si melts in the 0.09 < P < 26.5 at % composition range at different temperatures, ranging between 1423 and 1558 °C, using Knudsen effusion mass spectrometry [273]. However, both [256,257] considered the activity data of Zaitsev et al. [263] not reliable, because it predicts (Si-rich solid + Gas) two phase field in the 1125 to 1220 °C temperature range, which is not the case as could be proven by Safarian and Tangstad [255] using DTA measurements. The authors [256,257] gave higher weight to the experimental results of Miki et al. [239] in the thermodynamic modeling of P-Si system. The enthalpy of mixing of the P-Si liquid at 1427 °C [55] is shown in Figure 30a. The experimental results of activity measurements of P and Si in the P-Si liquid at 1427 °C [263] are given in Figure 30b. The activity calculations of Franke and Neuschütz [55] are consistent with these experimental results, therefore Franke and Neuschütz’ curves are adopted in this work.

### 5.9. The Sb-Si System

**Figure 31:**
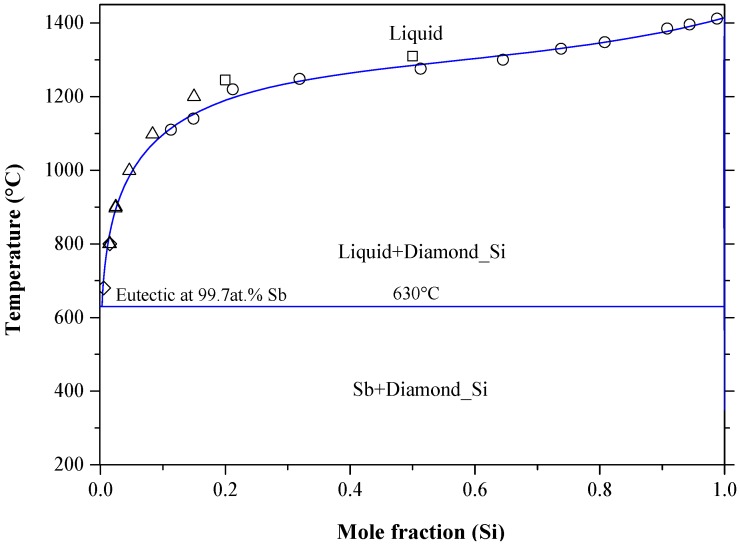
The calculated Sb-Si binary phase diagram by Wang et al. [277]; ○: [177]; □: [275]; Δ: [120]; ◇: [178].

**Figure 32:**
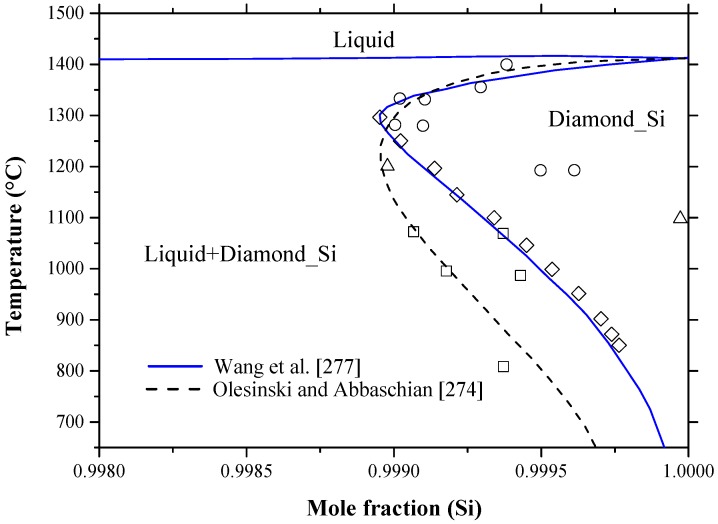
Magnified portion of the calculated Sb-Si phase diagram [277]; ----: [274]; Δ: [179]; ○: [276]; □: [118]; ◇: [278].

Antimony-silicon system seems to have no particular technological importance in its own right. However, understanding its thermodynamic properties is essential to model possible interactions between Si and Sb in electronic applications, such as in soldering processes [56]. The Sb-Si phase diagram was critically assessed by Olesinski and Abbaschian [274] based on the experimental results of [118,120,177,178,275,276]. Several authors calculated the Sb-Si phase diagram [101,120,274,277]. Wang et al. [277] reassessed the Sb-Si system, shown in Figure 31, to achieve compatibility among the binaries in the Au-Sb-Si system, using data from Dinsdale [237] for the pure elements and substitutional solution model to describe the liquid and solid solution phases. The optimized model parameters are listed in Table 17. The calculated liquid phase boundaries show very good agreement with most of the experimental data. Wang et al. [277] presented a modified Si-rich solvus line, different than that in [274], based on the experimental results of Nobili et al. [278]. The magnified part of the calculated Sb-Si phase diagram [277], near Si-rich side, associated with the experimental points is shown in Figure 32. Nobili et al. [278] measured the solid solubility of antimony in silicon by carrier density measurements on poly silicon films doped by ion implantation in the 850–1300 °C temperature range. Their measurements [278] proved to be accurate determination of the solubility values in this system. Therefore, the solvus line calculated by Wang et al. [277] is considered more accurate presentation of the Sb solubility in Si. 

**Figure 33:**
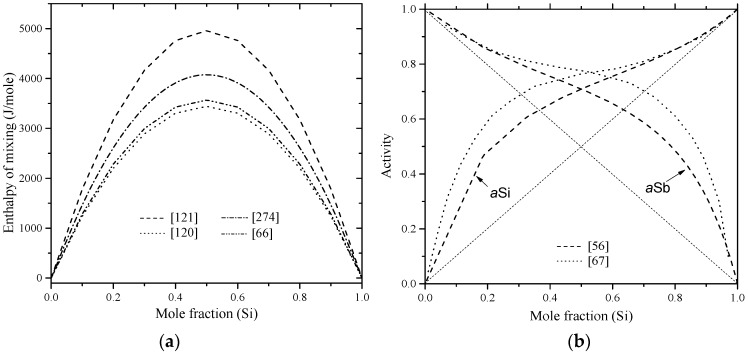
(**a**) Enthalpy of mixing of the Sb-Si liquid at 1427 °C; -----: [121]; ···· : [120]; ˗·˗·˗: [274]; ˗··˗··˗: [66] and (**b**) activity of Sb and Si in the Sb-Si liquid at 1477 °C [277]; ----: [56]; ····: [67].

The calculated Sb-Si phase diagram [277] exhibits a eutectic transformation at 0.3 at % Si and 630 °C. The solid solution of Si in Sb is considered negligible. The solid solubility of antimony in silicon showed retrograde behavior with a maximum of about 0.1 at % Sb at 1300 °C. The shape of the liquidus line indicates a likelihood of metastable miscibility gap with positive enthalpy of mixing [56] as could be seen in Figure 33a. The crystallographic data of the end-members of the Sb-Si system are listed in Table 2.

Antimony has a low diffusivity in silicon, compared to arsenic, for example, and it is known to diffuse via a vacancy mechanism. The diffusivity data of antimony in silicon was reviewed by Pichler [90] who expressed the intrinsic diffusivity of antimony by the equation: $D_{i}=40.9\times e^{\left(-4.158\;eV/kT\right)}\;{\mathrm{cm}}^{2}/s$.

Experimental thermodynamic properties of the Sb-Si system could not be found in the literature. The calculated thermodynamic properties, enthalpy of mixing and activity at 1477 °C, using different models are shown in Figure 33a,b, respectively. Thurmond [121] used the regular solution model and calculated the molar enthalpy of mixing based on the data of Williams [177]. However, the measured values by Williams [177] are considered not accurate, because low purity silicon was used. Olesinski and Abbaschian [274] modified the thermodynamic model parameters of [120,121] in order to be consistent with the experimental phase diagram data [118,120,177,178,275,276]. The liquid phase parameters were modified by Safarian et al. [66] in an attempt to keep the eutectic temperature and composition as that given by Olesinski and Abbaschian [274] at 629.7 °C and 0.3 at % Si. Yet, their calculated values were closer to those of [120]. The available activities of antimony and silicon [56,67], in Figure 33b, are inconsistent but show positive deviation from Raoult’s law, which is expected for such flat liquidus. Thus, experimental investigations of the enthalpy of mixing and activities are required to verify these thermodynamic properties.

### 5.10. The Tl-Si System

**Figure 34:**
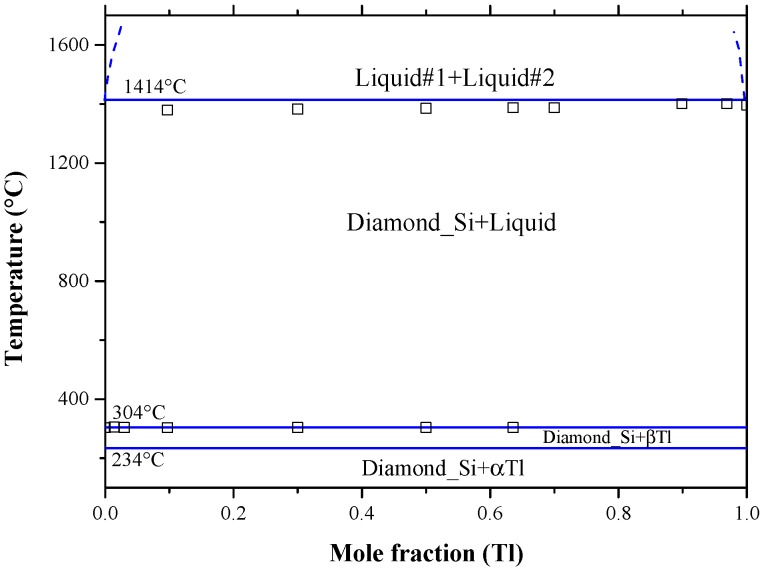
The Si-Tl phase diagram redrawn after Olesinski and Abbaschian [57]. □: [280].

According to Zhao et al. [279], thallium could be the most suitable impurity atom in crystalline Si solar sell industry, because it provides deep level of impurity photovoltaic effect, which improves the solar cell efficiency. Silicon and thallium are virtually immiscible. The liquid miscibility gap in the Si-Tl system was described by Savitskiy et al. [187] and Thurmond and Kowalchik [120], with some variations. Savitskiy et al. [187] reported the monotectic transformation temperature at 1414 °C, while it was reported by Thurmond and Kowalchik [120] to be 1387 °C. Olesinski and Abbaschian [57] have constructed a tentative Si-Tl phase diagram, shown in Figure 34, based on the thermal analysis of Tamaru [280] and Savitskiy et al. [187]. Later on, the Si-Tl phase diagram assessed by Olesinski and Abbaschian [57] was accepted by Predel [281]. The Si-Tl phase diagram shows a eutectic transformation near the melting point of thallium and a monotectic transformation near the melting point of silicon. The solubilities of both silicon and thallium are very infinitesimal [282]. However, Schmit and Scott [282] developed a novel method, including adding of a second metal (tin) to thallium, to grow silicon-doped semiconductor for 3–5 μm infrared radiation detector, which can operate above 77 K. 

The diffusion of thallium in silicon was reviewed by Hull [138], who described the diffusivity of thallium in the 1105–1360 °C temperature range by the equation *D = *16.5 *× e*^*(−*3.896 *eV/kT)*^ cm^2^/s, based on the work of Fuller and Ditzenberger [179]. However, Ghoshtagore [283] expressed the diffusion coefficient of thallium in silicon in the 1244–1338 °C temperature range as *D = *1.37 *× e*^*(−*3.896 *eV/kT)*^ cm^2^/s. The difference in these two sets of results could be due to oxidation/reduction reactions on the Si surface.

**Figure 35:**
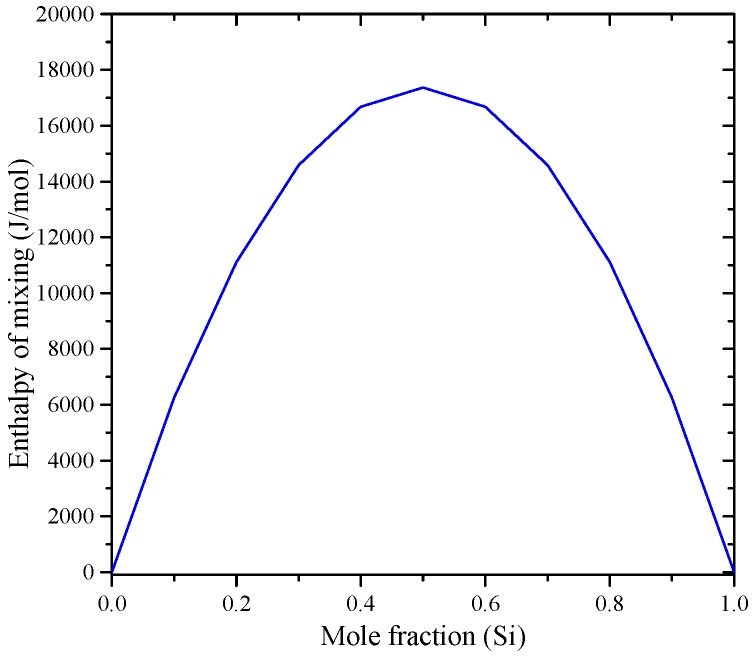
Calculated enthalpy of mixing of the Si-Tl liquid using thermodynamic coefficients from [120].

Very limited thermochemical data of the Si-Tl system could be found in the literature. Thurmond and Kowalchik [120] attempted to describe the Si-Tl liquid in terms of regular solution model. However, Olesinski and Abbaschian [57] considered the calculations of [120] speculative and inaccurate without providing enough justification. The enthalpy of mixing curve is shown in Figure 35 [120]. The positive enthalpy of mixing indicates the existence of a miscibility gap in the Si-Tl system, which agrees with the Si-Tl phase diagram shown in Figure 34. The model parameters of the liquid phase reported by [120] are listed in Table 18.

## 6. Discussion

It is concluded from the analyzed data in this paper that the melts of Si with Al, As, B and P (IIIA group) are exothermic and their activities deviate negatively from Raoult’s ideal solution, whereas the melts of Si with Bi, Ga, In, Sb and Tl (VA group) are endothermic in behavior and deviate positively from Raoult’s law. Furthermore, it can be said that the atoms of the VA group do not prefer to form homogenous mixtures with silicon upon melting. Positive enthalpy of mixing in this case is due to the de-mixing effect, by which Si and VA group atoms in Si-VA group mixtures favor to form bonds between similar atoms. This was observed in systems where the liquid curve tends to be flat indicating a metastable miscibility gap or when the liquid miscibility gap is stable.

**Figure 36:**
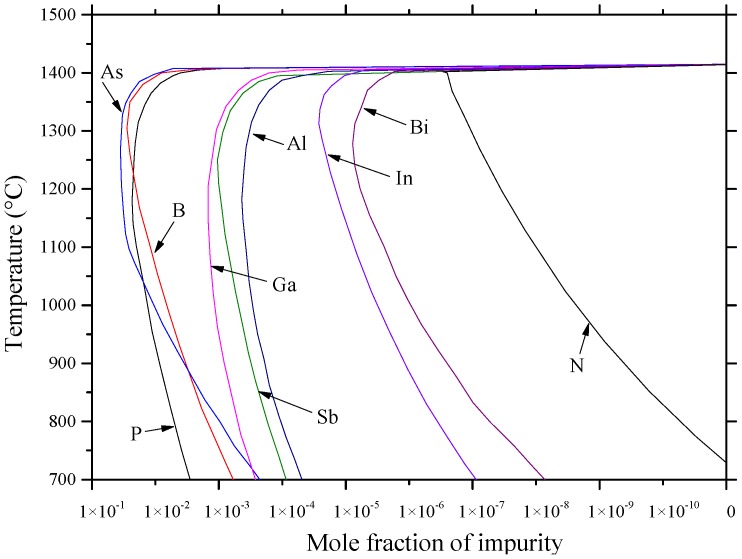
Summary of solid solubility of impurity atoms in silicon.

**Figure 37:**
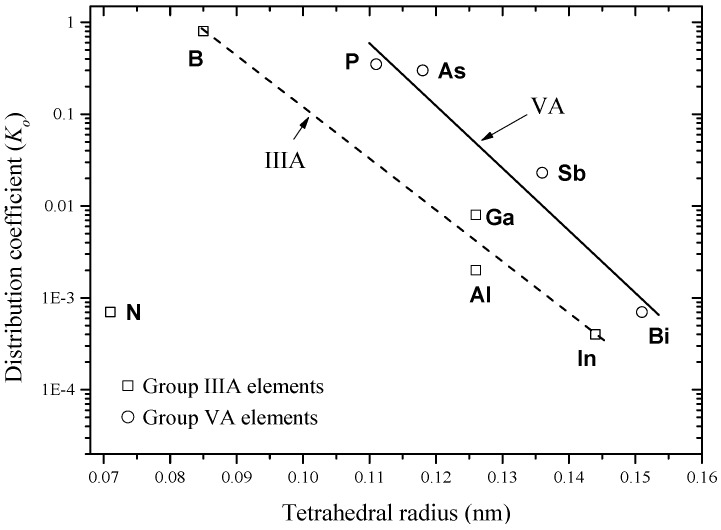
Distribution coefficients vs. tetrahedral radii of impurity atoms (except N) at the melting point of silicon.

In agreement with [105], Figure 36 shows a graphical summary of best representation of solid solubility of Al, As, B, Bi, Ga, In, N, P and Sb impurity atoms in silicon. No data regarding the solubility of thallium in silicon could be found in the literature. Boron, arsenic and phosphorus exhibit highest solid solubility in silicon than other impurity atoms included in this study, although they occupy substitutional positions in silicon lattice [26]. Here, the distribution coefficient, *K_o_* in Table 1, is chosen to compare the relative tendencies of various impurities to dissolve in solid silicon. B, As and P possess higher distribution coefficient values compared to other impurity atoms. Furthermore, Burton et al. [284] suggested a rough correlation between the distribution coefficient of an impurity atom and its tetrahedral radius at the melting point of silicon as demonstrated in Figure 37. The trend goes toward higher solubility as the radius of impurity atom decreases.

**Figure 38:**
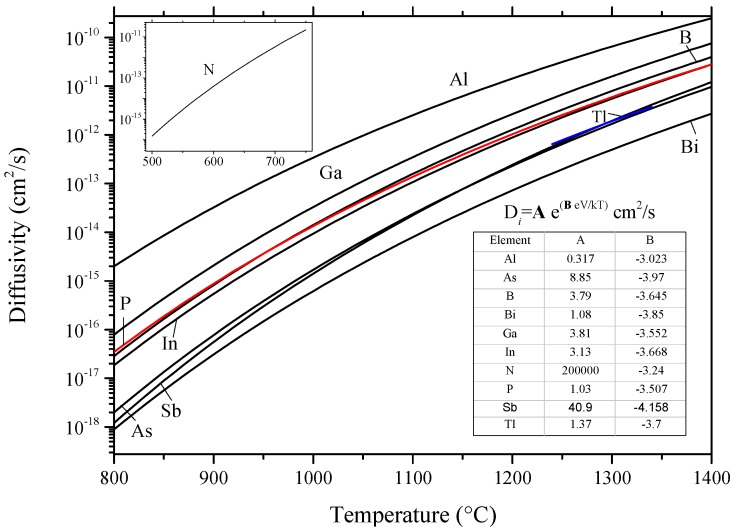
Summary of diffusivity of the studied elements in silicon. The parameters used in the diffusion equation are listed in the embedded table.

The temperature-dependent diffusion coefficients of the studied doping elements in silicon are plotted in Figure 38. This plot is useful for comparing relative diffusivities of several impurities. The parameters used in calculating these coefficients, listed in the embedded table, were taken from the literature [26,138,180]. The inset shows the low nitrogen diffusivity in the 500–750 °C temperature range. The diffusivity of co-diffusing atoms is greatly influenced by the size mismatch between silicon and the diffusing species [26]. The presence of size mismatch, irrespective to its value, leads to lattice strain, which may influence the diffusivity of the impurity atom [26]. Table 19 presents the relative atomic size of studied elements to silicon. It is concluded that Al is the fastest diffusing acceptor impurity in Si followed by Ga. Moreover, the fastest diffusing donor impurity in Si is P.

## 7. Summary

The success of producing or refining Si materials to high purity levels depends heavily on the availability and reliability of phase diagram, thermodynamic and diffusion data that serve as important tools in evaluating the effect of impurities on the phase equilibria in solar and electronic Si systems. In this work, ten Si-based binary phase diagrams, including Si with group IIIA elements (B, Al, Ga, In and Tl) and with group VA elements (N, P, As, Sb and Bi), have been reviewed. Each of these systems has been critically discussed in both aspects of phase diagram and thermodynamic properties. The available experimental data and thermodynamic parameters in the literature have been summarized and assessed thoroughly to provide comprehensive understanding of each system. Some systems were re-calculated to obtain the best evaluated phase diagram. As doping levels of solar and electronic silicon are of high technological importance, diffusion data have also been presented to serve as useful references on the properties, behavior and quantities of metal impurities in silicon. This paper is meant to bridge the understanding of phase diagrams with the information needed by the industry and research for development of solar-grade and electronic silicon, relying on the available information in the literature as well as our own analysis.

## Figures and Tables

**Table 1:** Distribution coefficient (*K_o_*) of studied elements in this work at melting point of Si.

Element	*K_o_* [28]	Element	*K_o_* [28]
Al	0.002	In	4 × 10^−4^
As	0.300	N^1^	7 × 10^−4^
B	0.800	P	0.350
Bi	7 × 10^−4^	Sb	0.023
Ga	0.008	Tl	-

^1^ *K_o_* for nitrogen was taken from [32].

**Table 2:** Crystallographic data of the solid phases in the binary systems covered in the current study.

System	Phase	Prototype	Lattice Parameters (nm)			Space Groupe Number	Space Group	Ref.
			*a*	*b*	*c*			
All systems	(Si)	C (diamond)	0.5430			227	*Fd-3m*	[38,39]
Al-Si	(Al)	Cu	0.4047			225	*Fm-3m*	[38,39]
As-Si	AsSi	AsSi	1.598	0.3668	0.953	12	*C2/m*	[39,40,41]
	As_2_Si	As_2_Ge	1.453	1.037	0.3728	55	*Pbam*	[39,42]
	As_2_Si ^1^	FeS_2_	6.0232			205	*Pa-3*	[41,43]
	(As)	αAs	0.3760		1.0682	166	*R-3m*	[39,41,43]
B-Si	β-B	β-B	1.101		2.3900	166	*R-3m h*	[39,44]
	B_3_Si	B_4_C	0.6319		1.2713	166	*R-3m h*	[39,45]
	B_6_Si	B_6_Si	1.439	1.827	0.988	58	*Pnnm*	[39,46]
	B_n_Si *n ≈ 23*	β-B	1.101		2.390	166	*R-3m*	[39,47,48]
Bi-Si	Bi	αAs	0.4546		1.1862	166	*R-3m*	[49,50]
Ga-Si	(αGa)	αGa	0.45192	0.76586	0.45258		*Cmca*	[39,51]
	(βGa)		0.27713	0.80606	0.33314	15	*C12/c1*	[39,51]
	(γGa)	γGa	1.0593	1.3523	0.5203	63	*Cmcm*	[39,51]
	(δGa)		0.909		1.702	177	*R-3m*	[39,51]
In-Si	(In)	In	0.4599		0.4947		*F4/mmm*	[52]
N-Si	αSi_3_N_4_	Si_3_N_4_	0.7818		0.5591	159	*P3*_1_*c*	[53]
	βSi_3_N_4_	Si_3_N_4_	0.7595		0.2902	173	*P6*_3_	[54]
	γSi_3_N_4_**^1^**	Fe_3_O_4_	0.7772			227	*Fd-3m*	[39]
P-Si	P(red)	P	0.9210	0.9250	2.2600	13	*P12/c1*	[39]
	SiP	SiP	0.3511	2.0488	1.3607	36	*Cmc2*_1_	[39,55]
	SiP_2_	GeAs_2_	1.397	1.008	0.3436	55	*Pbam*	[42]
	Si_6_P_2.5_	C_5_W_12_	0.616		1.317	162	*P-31m*	[39]
Sb-Si	Sb	αAs	0.4331		1.1374	166	*R-3m*	[39,56]
Si-Tl	αTl	Mg	0.3456		0.5526	194	*P6*_3_*/mmc*	[39,57]
	βTl	W	0.3882			229	*Im3m*	[39,57]

^1^ High-pressure phase.

**Table 3:** $D_{i}$ and $D_{ex}$ of aluminum in silicon.

Temperature (°C)	Intrinsic Diffusivity ^1^ $D_{i}$ (cm^2^/s)	Extrinsic Diffusivity ^1^ $D_{ex}$ (cm^2^/s)
900	1.9 × 10^−14^	1.2 × 10^−16^
1000	2.5 × 10^−13^	1.4 × 10^−15^
1100	2.3 × 10^−12^	1.0 × 10^−13^

^1^ Diffusivity values reported by [91].

**Table 4:** Optimized model parameters of the Al-Si system.

Phase	Excess Gibbs Energy Parameter (J/Mole)	Ref.
Liquid	^0^$L_{Al,Si}^{liq}=-11\;340.1-1.23394\;T$ ^1^$L_{Al,Si}^{liq}=-3\;530.93+1.35993\;T$ ^2^$L_{Al,Si}^{liq}=2\;265.39$	[59,98]
FCC_Al	^0^$L_{Al,Si}^{fcc}=-3\;143.78+0.39297\;T$	[59,98]
Diamond_Si	^0^$L_{Al,Si}^{Diamond}=113\;246.16-58.0001\;T$	This work

*T* is the absolute temperature in this table and throughout the paper.

**Table 5:** Comparison between the available As-Si phase diagram data.

Features	Olesinski and Abbaschian [112]	FTlite Database [124]
Si terminal solid solution	Retrograde behavior	Typical behavior
Si-rich eutectic	60 at % Si at 1097 °C	56 at % at 1108 °C
As-rich eutectic	9.8 at % Si at 797 °C	4.7 at % Si at 795 °C
As_2_Si peritectic temperature	977 °C	979 °C

**Table 6:** The enthalpy and entropy of formation of the As-Si intermetallic compounds (at 298 K and 1 atmospheric pressure).

Compound		Enthalpy of Formation kJ·(Mole·Atom)^−1^	Entropy of Formation (J/Mole·Atom·K)	Ref.
AsSi	Experimental	−5.4 ± 1.2	-	[140]
	Calculated	−114.0	-	[141]
	CALPHAD ^1^	−5.9	30.64	[124]
As_2_Si	Experimental	−3.7 ± 2.3	-	[140]
	Calculated	−89.0	-	[141]
	CALPHAD	−4.3	33.8	[124]

^1^ Calculations of phase diagram.

**Table 7:** Thermodynamic model parameters of the B-Si system.

Phase	Excess Gibbs Energy Parameter (J/Mole) [155]
Liquid	^0^$L_{B,Si}^{Liquid}=17\;631.92-1.76321\;T$ ^1^$L_{B,Si}^{Liquid}=-3\;526.99-0.3527\;T$
Diamond_Si	^0^$L_{B,Si}^{Dia-Si}=57\;978.16$
β-B	$G\;\left(B:Si\right)=-6\;160.245+0.6160245\;T+93.0\;GHSER_{B}+12.0\;GHSER_{Si}$ ^0^$L_{B:B,Si}^{\beta -B}=-725\;614+72.5614\;T$
B_3_Si	$G\;\left(B:Si:B\right)=112\;000+12.0\;GHSER_{B}+2.0\;GHSER_{Si}$ $G\;\left(B:Si:Si\right)=112\;000+6.0\;GHSER_{B}+8.0\;GHSER_{Si}$ ^0^$L_{B:Si:B,Si}^{B_{3}Si}=-2400\;475+240.0475\;T$
B_6_Si	$G\;\left(B:Si:B\right)=729\;824.4-72.98244\;T+258.0\;GHSER_{B}+23.0\;GHSER_{Si}$ $G\;\left(B:Si:Si\right)=5454\;560-545.456\;T+210.0\;GHSER_{B}+71.0\;GHSER_{Si}$ ^0^$L_{B:Si:B,Si}^{B_{6}Si}=-15715\;630+1571.563\;T$
B_n_Si	$G\;\left(B:Si:B\right)=-89\;819.86+8.981986\;T+69.0\;GHSER_{B}+GHSER_{Si}$ $G\;\left(B:Si:Si\right)=-176\;659.7+17.66597\;T+61.0\;GHSER_{B}+9.0\;GHSER_{Si}$ ^0^$L_{B:Si:B,Si}^{B_{n}Si}=-281\;573.6+28.15736\;T$

GHSER is Gibbs free energy values at reference state (1 atm and 298.15 K) for a pure element.

**Table 8:** The enthalpy and entropy of formation of the B-Si intermetallic compounds.

Compound	Enthalpy of Formation kJ·(Mole·Atom)^−1^	Entropy of Formation (J/Mole·Atom·K)	Ref.
B_3_Si	−6.150	−	[124,155]
	−11.119	−3.68	[153] CALPHAD
	−6.025	-	[169]
	−27.350	−16.18	[170]
B_4_Si	−18.16	-	[171]
B_6_Si	−4.230	-	[124,155]
	−21.186 ± 1.07	-	[142]
	−17.286 ± 6.85	−5.31	[148]
	−6.616	-	[169]
	−8.380	−1.43	[153] CALPHAD
	−17.457	-	[171]
B_14_Si	−3.825	-	[153] CALPHAD
B_n_Si, *n* = 15.67	−12.304 ± 1.37	-	[142]
B_n_Si, *n* = 36	−1.810	-	[124,155]
B_15_Si	−13.563 ± 4.84	−3.78	[148]
B_12_Si	−4.316	-	[169]

**Table 9:** Thermodynamic parameters of the liquid phase in Bi-Si system.

Phase	Excess Gibbs Energy Parameter (J/Mole)	Ref.
Liquid	^0^$L_{Bi,Si}^{liq}=46\;370+2.26\;T$	[50]
	^0^$L_{Bi,Si}^{liq}=62\;090-8.62\;T$	[120]

**Table 10:** Optimized model parameters of the Ga-Si system.

Phase	Excess Gibbs Energy Parameter (J/Mole)	Ref.
Liquid	^0^$L_{Ga,Si}^{liq}=14\;900+4.9\;T$	[51]

**Table 11:** Model parameters of the In-Si system.

Phase	Excess Gibbs Energy Parameter (J/mole)	Ref.
Liquid	^0^$L_{Ga,Si}^{liq}=45\;100+12.8\;T$	[52]

**Table 12:** Atom positions in the unit cell of Si_3_N_4_.

Phase	Symmetry	Pearson Symbol	Atom	Wyckoff Position	*x*	*y*	*z*
αSi_3_N_4_	Hexagonal	hP28	N1	6c	0.0424	0.3891	0.0408
			N2	6c	0.3169	0.3198	0.2712
			N3	2b	0.3333	0.6667	0.3649
			N4	2a	0.0000	0.0000	1.0000
			Si1	6c	0.0821	0.5089	0.3172
			Si2	6c	0.1712	0.2563	0.0274
βSi_3_N_4_	Hexagonal	hP14	N1	6c	0.0298	0.3294	0.2680
			N2	2b	0.3333	0.6667	1.0000
			Si	6c	0.7686	0.1744	0.2550
γSi_3_N_4_	Cubic	cF56	N	32e	0.8676	0.8676	0.8676
			Si1	8a	0	0	0
			Si2	16d	0.6250	0.6250	0.6250

**Table 13:** Optimized model parameters of the N-Si system.

Phase	Excess Gibbs Energy Parameter (J/Mole) [214]
Liquid	^0^$L_{N,Si}^{liq}=-87\;631-22.359\;T$
Si_3_N_4_	^0^$G_{N:Si}^{Si3N4}=-788\;513.009+733.225\;T-121.79\;T\ln\left(T\right)-0.020\;65\;T^{2}+1666\;886.4\;T^{-1}+6.9938\times 10^{-7}\;T^{3}$

**Table 14:** The enthalpy and entropy of formation of the N-Si intermetallic compounds.

Compound	Enthalpy of FormationkJ·(Mole·Atom)^−1^	Entropy of Formation(J/Mole·Atom·K)	Ref.
Si_3_N_4_	−103.400	−45.000	[216]

**Table 15:** Optimized model parameters of the P-Si system.

Phase	Excess Gibbs Energy Parameter (J/Mole) [257]
Gas	$G_{P}^{0,Gas}=327\;729.700-24.71\;T-20.67\;T\;\ln\left(T\right)-8.5\times 10^{-5}\;T^{2}+RT\;\ln\left(10^{-5}\times P\right)$ $G_{P2}^{0,Gas}=132\;066.5+27.5674\;T-36.30\;T\;\ln\left(T\right)-4.0\times 10^{-4}\;T^{2}+2.05\times 10^{5}\;T^{-1}+RT\;\ln\left(10^{-5}\times P\right)$ $G_{P4}^{0,Gas}=30\;174.8+275.871\;T-81.84\;T\;\ln\left(T\right)-3.4\times 10^{-4}\;T^{2}+6.70\times 10^{5}\;T^{-1}+RT\;\ln\left(10^{-5}\times P\right)$ $G_{Si}^{0,Gas}=444\;756.1-36.2676\;T-19.80\;T\;\ln\left(T\right)-5.05\times 10^{-4}\;T^{2}-1.05\times 10^{5}\;T^{-1}+RT\;\ln\left(10^{-5}\times P\right)$ $G_{Si2}^{0,Gas}=579\;613.8+1.2671\;T-34.50\;T\;\ln\left(T\right)+RT\;\ln\left(10^{-5}\times P\right)$ $G_{Si3}^{0,Gas}=619\;571.9+101.1376\;T-55.10\;T\;\ln\left(T\right)+RT\;\ln\left(10^{-5}\times P\right)$ $G_{Si4}^{0,Gas}=646\;000.0-292.8000\;T+RT\;\ln\left(10^{-5}\times P\right)$
Liquid	$L_{P,Si}^{0,Liq}=-20\;801.04+4.1172\;T$ $L_{P,Si}^{1,Liq}=13982.31-2.7877\;T$
Diamond_Si	$G_{P}^{\left(Si\right)}=30\;T+G_{P}^{0,white\_P}$ $L_{P,Si}^{\left(Si\right)}=-40\;498.48+21.8079\;T\;\left(Model\;I\right)$ $L_{P,Si}^{\left(Si\right)}=10\;594.1-25.17\;T\;\left(Model\;II\right)$
SiP	$G_{P:Si}^{0,SiP}=-77\;110.5+228.49\;T-38.34\;T\;\ln\left(T\right)-0.00544\;T^{2}+2.825\times 10^{5}\;T^{-1}$
SiP_2_	$G_{P:Si}^{0,SiP2}=-100\;809.0+389.8\;T-67.00\;T\;\ln\left(T\right)-0.00855\;T^{2}$
*P* is the pressure in Pascal	

**Table 16:** The enthalpy and entropy of formation of the P-Si intermetallic compound.

Compound	Enthalpy of Formation kJ·(Mole·Atom)^−1^	Entropy of Formation (J/Mole·Atom·K)	Ref.
SiP	−61.118	15.016	[55]
	−71.128	-	[271]
	−61.923	32.635	[124,242]
	−63.300	34.735	[256]
	−63.300	34.740	[257]
	−63.220	29.400	[272]
SiP_2_	−80.070	64.050	[257]
	−79.300	67.000	[256]
	−84.300	69.900	[270]

**Table 17:** Optimized model parameters of the Sb-Si system [277].

Phase	Excess Gibbs Energy Parameter (J/Mole)	Ref.
Liquid	°$L_{Sb,Si}^{liq}=15\;463.82+4.805\;T$	[277]
Diamond_Si	°$L_{Sb,Si}^{Diamond}=67\;795.81$	[277]
Sb	Obtained from SGTE database	[237]

**Table 18:** Model parameters of the Si-Tl system.

Phase	Excess Gibbs Energy Parameter (J/Mole)	Ref.
Liquid	^0^$L_{Si,Tl}^{liq}=69\;500+15.9\;T$	[120]

**Table 19:** Size of impurity atom relative to silicon.

Group	Element	Tetrahedral Radius (nm)	Size Ratio to Si	Ref.
III A Acceptors	B	0.085	0.72	[284]
	Al	0.126	1.07	[26,284]
	Ga	0.126	1.07	[26,284]
	In	0.144	1.22	[26,284]
	Tl	0.144	1.22	[284]
V A Donors	N	0.071	0.60	[285]
	P	0.111	0.94	[284]
	As	0.118	1.00	[284]
	Sb	0.136	1.15	[26]
	Bi	0.151	1.28	[284]

## Abbreviations

Al	Aluminum
As	Arsenic
a-Si	Amorphous silicon
B	Boron
Bi	Bismuth
Calphad	Calculations of phase diagram
CdTe	Cadmium telluride
CIGS	Copper indium gallium diselenide
*C_m_*	Maximum solid solubility
CO_2_	Carbon dioxide
c-Si	Crystalline silicon
*C_sat_*	Saturation concentration
Cu(In,Ga)Se_2_	Copper indium gallium diselenide
*D*	Diffusivity
*D*_0_	Temperature-independent pre-exponential factor
*D_eff_*	Effective diffusivity
*D_ex_*	Extrinsic diffusivity
*D_i_*	Intrinsic diffusivity
DSSC	Dye-sensitized solar cells
DTA	Differential thermal analysis
EPMA	Electron probe microanalyzer
Ga	Gallium
GHSER	Gibbs free energy values at reference state (1 atm and 298.15 K) for a pure element
ICP	Inductively coupled plasma
In	Indium
IR	Infra-red
*k*	Boltzmann constant
*K_o_*	Distribution coefficient
N	Nitrogen
P	Phosphorus
*P*	Pressure
ppb	Particle per billion
PV	Photovoltaic
*Q_d_*	Activation energy
*R*	Universal gas constant
Sb	Antimony
Si	Silicon
Si:As	Arsenic-doped-silicon
Si:Bi	Bismuth-doped-silicon
Si:P	Phosphorus-doped-silicon
*T*	Absolute temperature
TEM	Transition electron microscopy
TFSC	Thin film solar cells
TG	Thermogravimetric
TGMZ	True gradient melting zone
Tl	Thallium
WDS	Wavelength dispersive spectroscopy
*X_m_*	Maximum molar solid solubility
XRD	X-ray diffraction

## References

[1] Luque A., Hegedus S., Luque A., Hegedus S. (2010). Handbook of Photovoltaic Science and Engineering.

[2] Anwar S., Efstathiadis H., Qazi S. (2013). Handbook of Research on Solar Energy Systems and Technologies.

[3] Singh V.K., Giribabu L. (2013). Photovoltaic—A Review of the Solar Cell Generation. J. Innov. Electron. Commun..

[4] Sovacool B.K. (2008). The Costs of Failure: A Preliminary Assessment of Major Energy Accidents, 1907–2007. Energy Policy.

[5] Hollangel E., Fujita Y. (2013). The Fukushima Disaster—Systemic Failures as the Lack of Resilience. Nucl. Eng. Technol..

[6] Parida B., Iniyan S., Goic R. (2011). A Review of Solar Photovoltaic Technologies. Renew. Sustain. Energy Rev..

[7] Becquerel A.E. (1841). Mémoire Sur Les Effets Électriques Produits Sous L’influence Des Rayons Solaires. Ann. Phys. Chem..

[8] Becquerel A.E. (1839). Recherches Sur Les Effets de La Radiation Chimique de La Lumiere Solaire Au Moyen Des Courants Electriques. CR Acad. Sci..

[9] Badawy W.A. (2015). A Review on Solar Cells from Si-Single Crystals to Porous Materials and Quantum Dots. J. Adv. Res..

[10] Chopra K.L., Paulson P.D., Dutta V. (2004). Thin-Film Solar Cells: An Overview. Prog. Photovolt. Res. Appl..

[11] Green M.A. (2007). Thin-Film Solar Cells: Review of Materials, Technologies and Commercial Status. J. Mater. Sci. Mater. Electron..

[12] Kerr M.J., Cuevas A., Campbell P. (2003). Limiting Efficiency of Crystalline Silicon Solar Cells due to Coulomb-Enhanced Auger Recombination. Prog. Photovolt. Res. Appl..

[13] Tsuo Y.S., Wang T.H., Ciszek T.F. (1999). Crystalline-Silicon Solar Cells for the 21st Century.

[14] Green M.A. (1999). Limiting Efficiency of Bulk and Thin-Film Silicon Solar Cells in the Presence of Surface Recombination. Prog. Photovolt. Res. Appl..

[15] Aberle A.G. (2000). Surface Passivation of Crystalline Silicon Solar Cells: A Review. Prog. Photovolt. Res. Appl..

[16] Tiedje T., Yablonovitch E., Cody G.D., Brooks B.G. (1984). Limiting Efficiency of Silicon Solar Cells. IEEE Trans. Electron. Devices.

[17] Chroneos A., Sgourou E.N., Londos C.A., Schwingenschlögl U. (2015). Oxygen Defect Processes in Silicon and Silicon Germanium. Appl. Phys. Rev..

[18] Sgourou E.N., Timerkaeva D., Londos C.A., Aliprantis D., Chroneos A., Caliste D., Pochet P. (2013). Impact of Isovalent Doping on the Trapping of Vacancy and Interstitial Related Defects in Si. J. Appl. Phys..

[19] Wang H., Chroneos A., Londos C.A., Sgourou E.N., Schwingenschlögl U. (2013). A-Centers in Silicon Studied with Hybrid Density Functional Theory. Appl. Phys. Lett..

[20] Van der Zwaan B., Rabl A. (2003). Prospects for PV: A Learning Curve Analysis. Sol. Energy.

[21] Jia G. (2010). Characterization of Electrical and Optical Properties of Silicon Based Materials.

[22] Graff K. (1995). Metal Impurities in Silicon-Device Fabrication.

[23] Coletti G. (2011). Impurities in Silicon and Their Impact on Solar Cell Performance.

[24] Fahey P.M., Griffin P.B., Plummer J.D. (1989). Point Defects and Dopant Diffusion in Silicon. Rev. Mod. Phys..

[25] Taylor W., Gosele U., Tan T.Y. Present Understanding of Point Defect Parameters and Diffusion in Silicon: An Overview. Proceedings of the Third International Symposium on Process Physics and Modeling in Semiconductor Technology.

[26] Scotten W.J. (2000). Diffusion in Silicon.

[27] Tang K., Øvrelid E.J., Tranell G., Tangstad M. (2009). A Thermochemical Database for the Solar Cell Silicon Materials. Mater. Trans..

[28] O’Mara W., Herring R.B., Hunt L.P. (2007). Handbook of Semiconductor Silicon Technology.

[29] Gudmundsen R.A., Maserjian J. (1957). Semiconductor Properties of Recrystallized Silicon in Aluminum Alloy Junction Diodes. J. Appl. Phys..

[30] Mckelvey A.L. (1996). Retrograde Solubility in Semiconductors. Metall. Mater. Trans. A.

[31] Khan M.I., Mostafa A.O., Aljarrah M., Essadiqi E., Medraj M. (2014). Influence of Cooling Rate on Microsegregation Behavior of Magnesium Alloys. J. Mater..

[32] Yatsurugi Y., Akiyama N., Endo Y., Nozaki T. (1973). Concentration, Solubility, and Equilibrium Distribution Coefficient of Nitrogen and Oxygen in Semiconductor Silicon. J. Electrochem. Soc..

[33] Hall R.N., Soltys T.J. (1971). High Purity Germanium for Detector Fabrication. IEEE Trans. Nucl. Sci..

[34] Fischler S. (1962). Correlation between Maximum Solid Solubility and Distribution Coefficient for Impurities in Ge and Si. J. Appl. Phys..

[35] Gumaste J.L., Mohanty B.C., Galgali R.K., Syamaprasad U., Nayak B.B., Singh S.K., Jena P.K. (1987). Solvent Refining of Metallurgical Grade Silicon. Sol. Energy Mater..

[36] Tafaghodikhajavi L. (2015). Thermodynamics of Impurity Removal in Solvent Refining of Silicon.

[37] Pizzini S. (2015). Physical Chemistry of Semiconductor Materials and Processes.

[38] Predel B. (2002). Al-Si. Binary Systems. Part 1_Elements and Binary Systems from Ag-Al to Au-Tl.

[39] Villars P., Cenzual K. (2009/2010). Pearson’s Crystal Data—Crystal Structure Database for Inorganic Compounds (on CD-ROM).

[40] Beck C.G. (1966). Crystallography of SiP and SiAs Single Crystals and of SiP Precipitates in Si. J. Appl. Phys..

[41] Han Q., Schmid-Fetzer R. (1993). Phase Equilibria in Ternary Ga-As-M Systems (M = W, Re, Si). J. Mater. Sci. Mater. Electron..

[42] Wadsten T., Vikan M., Krohn C., Nilsson Å., Theorell H., Blinc R., Paušak S., Ehrenberg L., Dumanović J. (1967). The Crystal Structures of SiP_2_, SiAs_2_, and GeP. Acta Chem. Scand..

[43] Massalski T.B., Okamoto H., Subramanian P.R., Kacprzak L. (1990). Binary Alloy Phase Diagrams.

[44] Wu J., Ma W., Tang D., Jia B., Yang B., Liu D., Dai Y. (2012). Thermodynamic Description of Si-B Binary System. Procedia Eng..

[45] Magnusson B., Brosset C. (1962). The Crystal Structure of В_2.8_Si. Acta Chem. Scand..

[46] Cline C.F. (1959). An Investigation of the Compound Silicon Boride (SiB_6_]). J. Electrochem. Soc..

[47] Giese R.F., Matkovich V.I., Economy J. (1965). The Crystal Structure of YB 4. Z. Krist..

[48] Korniyenko K. (2009). Boron—Carbon—Silicon. Landolt-Börnstein-Group IV Physical Chemistry 11E1 (Refractory Metal Systems).

[49] Franke P., Neuschütz D. (2004). Bi-Si. Binary Systems. Part 2: Elements and Binary Systems from B—C to Cr—Zr.

[50] Olesinski R.W., Abbaschian G.J. (1985). The Bi-Si (Bismuth-Silicon) System. Bull. Alloy Phase Diagr..

[51] Olesinski R.W., Kanani N., Abbaschian G.J. (1985). The Ga-Si (Gallium-Silicon) System. Bull. Alloy Phase Diagr..

[52] Olesinski R.W., Kanani N., Abbaschian G.J. (1985). The In-Si (Indium-Silicon) System. Bull. Alloy Phase Diagr..

[53] Kato K., Inoue Z., Kijima K., Kawada I., Tanaka H., Yamane T. (1975). Structural Approach to the Problem of Oxygen Content in Alpha Silicon Nitride. J. Am. Ceram. Soc..

[54] Grün R. (1979). The Crystal Structure of β-Si_3_N_4_: Structural and Stability Considerations between α- and β-Si_3_N_4_. Acta Crystallogr. Sect. B Struct. Crystallogr. Cryst. Chem..

[55] Franke P., Neuschütz D. (2006). P-Si. Binary Systems. Part 4: Binary Systems from Mn-Mo to Y-Zr.

[56] Franke P., Neuschütz D. (2006). Sb-Si. Binary Systems. Part 4: Binary Systems from Mn-Mo to Y-Zr.

[57] Olesinski R.W., Abbaschian G.J. (1985). The Si-Tl (Silicon-Thallium) System. Bull. Alloy Phase Diagr..

[58] Murray J.L., McAlister A.J. (1984). The Al-Si (Aluminum-Silicon) System. Bull. Alloy Phase Diagr..

[59] Liang Y., Guo C., Li C., Du Z. (2009). A Thermodynamic Description of the Al-Cr-Si System. J. Phase Equilib. Diffus..

[60] Dix E.H., Heath A.C. (1928). Equilibrium Relations in Aluminum-Silicon and Aluminum-Iron-Silicon Alloys of High Purity. Trans. AIME.

[61] Losana L., Stratta R. (1931). Solid Solubility of Silicon in Aluminum in the Solid State, in Italian. Metall. Ital..

[62] Durer A. (1940). Determination of Solvus Curves by Dilatometric Measurements. Z. Metallkd..

[63] Glazov V. (1961). Plotting of the solidus lines by measuring microhardness. Izv. Akad. Nauk SSSR Otd. Teckhn. Nauk. Met. Topl..

[64] Drits M.Y., Kadaner E.S., Kuz’mina V.I. (1968). Solubility of Silicon and Zirconium in Aluminium. Izv. Akad. Nauk SSR Met..

[65] Mondolfo L.F. (1976). Aluminum Alloys: Structure and Properties.

[66] Safarian J., Kolbeinsen L., Tangstad M. (2011). Liquidus of Silicon Binary Systems. Metall. Mater. Trans. B.

[67] Safarian J., Kolbeinsen L., Tangstad M. (2012). Thermodynamic Activities in Silicon Binary Melts. J. Mater. Sci..

[68] Navon D., Chernyshov V. (1957). Retrograde Solubility of Aluminum in Silicon. J. Appl. Phys..

[69] Miller R.C., Savage A. (1956). Diffusion of Aluminum in Single Crystal Silicon. J. Appl. Phys..

[70] Yoshikawa T., Morita K. (2003). Solid Solubilities and Thermodynamic Properties of Aluminum in Solid Silicon. J. Electrochem. Soc..

[71] Nishi Y., Kang Y., Morita K. (2010). Control of Si Crystal Growth during Solidification of Si-Al Melt. Mater. Trans..

[72] Martin J.W. (2007). Concise Encyclopedia of the Structure of Materials.

[73] Soma T., Funayama Y., Kagaya H.-M. (1990). Solid Solubility of Silicon and Germanium in Aluminium under Pressure. J. Mater. Sci..

[74] Fujishiro I., Mii H., Senoo M., Akao M. (1971). High-Pressure Phase Diagram of Al-Si System. J. Soc. Mater. Eng. Jpn..

[75] Senoo M., Mii H., Fujishiro I., Fujikawa T. (1976). Precise Measurements of Lattice Compression of Al, Si and Al-Si Alloys by High Pressure X-Ray Diffractometry. Jpn. J. Appl. Phys..

[76] Fraenkel W. (1908). Silicon–aluminum alloys. Z. Anorg. Chem..

[77] Roberts C.E. (1914). CXXIX.—The Alloys of Aluminium and Silicon. J. Chem. Soc. Trans..

[78] Gwyer A.G.C., Phillips H.W.L. (1927). Alloys of Aluminium with Silicon and Iron. J. Inst. Met..

[79] Broniewski W., Smialowski M. (1932). On the Al-Si Alloys. Rev. Met..

[80] Matsuyama K. (1934). Ternary Diagram of the Al-Cu-Si System. Kinzoku No Kenkyu.

[81] Craighead C.M., Cawthorne E.W., Jaffee R.I. (1955). Solution Rate of Solid Aluminum in Molten Al-Si Alloy. Trans. AIME.

[82] Berthon O., Petot-Ervas G., Petot C., Desres P. (1969). Thermodynamics of Aluminium-Silicon Alloys Containing 3–5 at% of Silicon. CR Acad. Sci. Paris.

[83] Kobayashi K., Shingu P.H., Kanbara H., Ozaki R. (1976). The Role of the Primary Phase on Eutectic Solidification of Al-Si Alloys. Trans. Jpn. Inst. Met..

[84] Kobayashi K., Shingu R.H., Ozaki R. (1976). The Role of the Primary Phase on Eutectic Solidification of Al-Si Alloys. Scr. Met..

[85] Girault B., Chevrier F., Joullie A., Bougnot G. (1977). Liquid Phase Epitaxy of Silicon at Very Low Temperatures. J. Cryst. Growth.

[86] Singer A.R.E., Jennings P.H. (1947). Hot-Shortness of the Aluminium-Silicon Alloys of Commercial Purity. J. Inst. Met..

[87] Mey S., Hack K. (1986). A Thermodynamic Evaluation of the Si-Zn, Al-Si and Al-Si-Zn Systems. Z. Met..

[88] Dörner P., Henig E.-T., Krieg H., Lukas H.L., Petzow G. (1980). Optimization and Calculation of the Binary System Al-Si. Calphad.

[89] Lozovskii V.N., Udyanskaya A.I. (1968). Solubility of Aluminum in Silicon Single Crystals. Inorg. Mater..

[90] Pichler P. (2004). Intrinsic Point Defects, Impurities, and Their Diffusion in Silicon.

[91] Krause O., Ryssel H., Pichler P. (2002). Determination of Aluminum Diffusion Parameters in Silicon. J. Appl. Phys..

[92] Galvagno G., Scandurra A., Raineri V., Rimini E., La Ferla A., Sciascia V., Frisina F., Raspagliesi M., Ferla G. (1993). Implants of Aluminum into Silicon. Nucl. Instrum. Methods Phys. Res. Sect. B Beam Interact. Mater. Atoms.

[93] Fisher D.J. (2010). Diffusivity in Silicon 1953 to 2009. Defect Diffus. Forum.

[94] Tang K., Øvrelid E.J., Tranell G., Tangstad M. (2009). Critical Assessment of the Impurity Diffusivities in Solid and Liquid Silicon. JOM.

[95] Chakraborti N., Lukas H.L. (1992). Thermodynamic Optimization of the Mg-Al-Si Phase Diagram. Calphad.

[96] Feufel H., Gödecke T., Lukas H.L., Sommer F. (1997). Investigation of the Al-Mg-Si System by Experiments and Thermodynamic Calculations. J. Alloy. Compd..

[97] Du Y., Schuster J.C., Liu Z.-K., Hu R., Nash P., Sun W., Zhang W., Wang J., Zhang L., Tang C. (2008). A Thermodynamic Description of the Al-Fe-Si System over the Whole Composition and Temperature Ranges via a Hybrid Approach of CALPHAD and Key Experiments. Intermetallics.

[98] Gröbner J., Lukas H.L., Aldinger F. (1996). Thermodynamic Calculation of the Ternary System Al-Si-C. Calphad.

[99] Tang Y., Du Y., Zhang L., Yuan X., Kaptay G. (2012). Thermodynamic Description of the Al-Mg-Si System Using a New Formulation for the Temperature Dependence of the Excess Gibbs Energy. Thermochim. Acta.

[100] Desai P.D. (1987). Thermodynamic Properties of Selected Binary Aluminum Alloy Systems. J. Phys. Chem. Ref. Data.

[101] Hansen S.C., Loper C.R. (2000). Effect of Antimony on the Phase Equilibrium of Binary Al-Si Alloys. Calphad.

[102] Bros J.P., Eslami H., Gaune P. (1981). Thermodynamics of Al-Si and Al-Ge-Si Liquid Alloys: Enthalpies of Formation by High Temperature Calorimetry. Ber. Bunsenges. Phys. Chem..

[103] Gizenko N.V., Emlin B.I., Kilesso S.N., Zav’yalov A.L. (1983). Heats of Formation of Molten Aluminum-silicon Alloys. Izv. Akad. Nauk SSR Met..

[104] Rostovtsev S.T., Khitrik S.I. (1971). Activity of Components in Silicon-aluminum, Silicon-manganese, and Silicon-chromium Alloys. Izv. Vyssh. Ucheb. Zaved. Met..

[105] Tang K., Øvrelid E.J., Tranell G., Tangstad M. (2009). Thermochemical and Kinetic Databases for the Solar Cell Silicon Materials. Adv. Mate. Res..

[106] Batalin G.I., Beloborodova E.A. (1971). An Investigation of the Thermodynamic Properties of Al-Si Melts. Izv. Akad. Nauk SSSR Met..

[107] Chatillon C., Allibert M., Pattoret A. (1975). Thermodynamic Study by Mass Spectrometry of Aluminum-Silicon Alloys From 1000 to 1700 K. High Temp. High Press..

[108] Loseva A., Al’mukhamedov A., Tyumentsev V., Luzhnova M. (1977). Thermodynamic Properties of Liquid Al-Si Alloys. Russ. J. Phys. Chem..

[109] Gokcen N.A. (1989). The As (Arsenic) System. Bull. Alloy Phase Diagr..

[110] Klement W., Jayaraman A., Kennedy G.C. (1963). Phase Diagrams of Arsenic, Antimony, and Bismuth at Pressures up to 70 Kbars. Phys. Rev..

[111] Jayaraman A., Klement W.J., Newton R.C., Kennedy G.C. (1962). High-Pressure Investigations of Group III Elements. Part I. Fusion Curves and Polymorphic Transitions of Aluminum, Gallium, Indium, and Thallium at High Pressures. Part II. Phase Transformations in Uranium at High Pressures.

[112] Olesinski R.W., Abbaschian G.J. (1985). The As-Si (Arsenic-Silicon) System. Bull. Alloy Phase Diagrams.

[113] Klemm W., Pirscher P. (1941). Uber Siliciumarsenide. Z. Anorg. Allg. Chem..

[114] Ugay Y.A., Miroshnichenko S.N., Goncharov E.G. (1974). Investigation of the pTx Diagram of the Si-As System. Izv. Akad. Nauk SSSR Neorg. Mat..

[115] Ugay Y.A., Goncharov E.G., Gladyshev N.F., Popov A.E., Inozemtseva N.Y. (1981). Tensimetric Study of the Si-As System. Fiz. Khim. Prots. Polyprovod. Poverkh.

[116] Belousov V.I. (1979). Calculation of the Activity Coefficient of Arsenic in Single Crystal Silicon. Russ. J. Phys. Chem..

[117] Sandhu J.S., Reuter J.L. (1971). Arsenic Source Vapor Pressure Kinetics and Capsule Diffusion. IBM J. Res. Dev..

[118] Trumbore F.A. (1960). Solid Solubilities of Impurity Elements in Germanium and Silicon. Bell Syst. Tech. J..

[119] Donohue P.C., Siemons W.J., Gillson J.L. (1968). Preparation and Properties of Pyrite-Type SiP_2_ and SiAs_2_. J. Phys. Chem. Solids.

[120] Thurmond C.D., Kowalchik M. (1960). Germanium and Silicon Liquidus Curves. Bell Syst. Tech. J..

[121] Thurmond C.D. (1953). Equilibrium Thermochemistry of Solid and Liquid Alloys of Germanium and of Silicon. I. The Solubility of Ge and Si in Elements of Groups III, IV and V. J. Phys. Chem..

[122] Jordan A.S., Weiner M.E. (1974). Calculation of the Liquidus Isotherms and Component Activities in the Ga-As-Si and Ga-P-Si Ternary Systems. J. Electrochem. Soc..

[123] Sudavtsova V.S., Batalin G.I. (1977). Calculation of the Activities of the Components of Molten Metal-Si Alloys from the Phase Diagrams. Ukr. Khim. Zh..

[124] FACTSAGE (2001). Integrated Thermodynamic Databank System.

[125] Fair R.B., Weber G.R. (1973). Effect of Complex Formation on Diffusion of Arsenic in Silicon. J. Appl. Phys..

[126] Ohkawa S., Nakajima Y., Fukukawa Y. (1975). Arsenic Diffusion into Silicon from Elemental Source. Jpn. J. Appl. Phys..

[127] Angelucci R., Armigliato A., Landi E., Nobili D., Solmi S. Equilibrium Solubility of Arsenic and Antimony in Silicon. Proceedings of the 17th European Solid State Device Research Conference (ESSDERC).

[128] Nobili D., Solmi S., Parisini A., Derdour M., Armigliato A., Moro L. (1994). Precipitation, Aggregation, and Diffusion in Heavily Arsenic-Doped Silicon. Phys. Rev. B.

[129] Nobili D., de Cogan D. (1999). Solubility of B, Al, Ga, In, Tl, P, As an Sb in c-Si. Prop. Cryst. Silicon INSPEC Inst. Electr. Eng. Lond..

[130] Fair R.B., Tsai J.C.C. (1975). The Diffusion of Ion-Implanted Arsenic in Silicon. J. Electrochem. Soc..

[131] Miyamoto N., Kuroda E., Yoshida S. (1974). The Behavior of Arsenic in Silicon During Heat Treatment. J. Jpn. Soc. Appl. Phys. Suppl..

[132] Pandey K.C., Erbil A., Cargill G.S., Boehme R.F., Vanderbilt D. (1988). Annealing of Heavily Arsenic-Doped Silicon: Electrical Deactivation and a New Defect Complex. Phys. Rev. Lett..

[133] Ventzek P.L.G., Kweon K.E., Ueda H., Oka M., Sugimoto Y., Hwang G.S. (2014). Formation, Nature, and Stability of the Arsenic-Silicon-Oxygen Alloy for Plasma Doping of Non-Planar Silicon Structures. Appl. Phys. Lett..

[134] Nobili D., Solmi S. (2005). Features of Arsenic Clusters in Silicon. Phys. Status Solidi.

[135] Moynagh P.B., Rosser P.J. (1989). Quantification of Diffusion Mechanisms of Boron, Phosphorus, Arsenic, and Antimony in Silicon. ESSDERC 1989.

[136] Kim Y., Massoud H.Z., Fair R.B. (1989). The Effect of Ion-Implantation Damage on Dopant Diffusion in Silicon during Shallow-Junction Formation. J. Electron. Mater..

[137] Fair R., Wang F.F.Y. (1981). Concentration Profiles of Diffusion Dopants in Silicon. Impurity Doping Processes in Silicon.

[138] Hull R. (1999). Properties of Crystalline Silicon.

[139] Pelton A.D. (1988). On the Slopes of Phase Boundaries. Metall. Trans. A.

[140] Fitzner K., Kleppa O.J. (1996). Direct Synthesis Calorimetry of Some Binary Alloys in the Systems Si-As, Ge-As and Sn-As. J. Alloy. Compd..

[141] Niessen A.K., de Boer F.R., Boom R., de Châtel P.F., Mattens W.C.M., Miedema A.R. (1983). Model Predictions for the Enthalpy of Formation of Transition Metal Alloys II. Calphad.

[142] Zaitsev A.I., Kodentsov A.A. (2001). Thermodynamic Properties and Phase Equilibria in the Si-B System. J. Phase Equilib..

[143] Olesinski R.W., Abbaschian G.J. (1984). The B-Si (Boron-Silicon) System. Bull. Alloy Phase Diagr..

[144] Brosset C., Magnusson B. (1960). The Silicon-Boron System. Nature.

[145] Samsonov G.V., Sleptsov V.M. (1962). Forefront Version of the Phase Diagram in the Boron-Silicon System. Acad. Sci. UK SSR.

[146] Hesse J. (1968). Loeslichkeit Und Ausscheidungskinetik von Bor in Polykristallinem Silizium. Z. Met..

[147] Male G., Salanoubat D. (1981). The Existence of a Rich Boron Phase in the Boron-Silicon System. Rev. Int. Ht. Temp. Refract..

[148] Armas B., Chatillon C., Allibert M. (1981). Determination by Differential Mass Spectrometry of Activities in the Solid Silicon-Boron System. Rev. Int. Ht. Temp. Refract..

[149] Barin I., Knacke O., Kubaschewski O. (1977). Thermochemical Properties of Inorganic Substances.

[150] Esin Y.O., Kolesnikov S.P., Baev B.M., Ermakov A.F. (1978). Entalpij Obrazovanya Zhidkikh Splavov Kremnya S Borom (Ethalpies Formation of Liquid Alloys of Silicon with Boron), Tezisy Nauchn. Soobshch. Vses. Konf. Str. Svoistvam Met. Shlakovykh Rasplav..

[151] Li J., Goto T., Hirai T. (1998). Thermoelectric Properties of SiB1_4_-SiB_6_ Composites Prepared by Arc Melting and Annealing. J. Jpn. Soc. Powder Powder Metall..

[152] Ettmayer P., Horn H.C., Schwetz K.A. (1970). Untersuchungen Im System Silicium-Bor Mit Hilfe Der Elektronenstarhl-Mikroanalyse. Mikrochim. Acta.

[153] Dirkx R.R., Spear K.E. (1987). Optimization of Thermodynamic Data for Silicon Borides. Calphad.

[154] Okamoto H. (2005). B-Si (Boron-Silicon). J. Phase Equilib. Diffus..

[155] Fries S., Lukas H.L., Ansara I., Dinsdale A.T., Rand M.H. (1998). System B-Si. COST 507. Thermochemical Database for Light Metal Alloys.

[156] Seifert H.J., Aldinger F. (2002). Phase Equilibria in the Si-B-C-N System. High Performance Non-Oxide Ceramics I.

[157] Vick G.L., Whittle K.M. (1969). Solid Solubility and Diffusion Coefficients of Boron in Silicon. J. Electrochem. Soc..

[158] Van Hung V., Hong P.T.T., Van Khue B. (2010). Boron and Phosphorus Diffusion In Silicon: Interstitial, Vacancy and Combination Mechanisms. Proc. Natl. Conf. Theor. Phys..

[159] Nichols C.S., Van de Walle C.G., Pantelides S.T. (1989). Mechanisms of Dopant Impurity Diffusion in Silicon. Phys. Rev. B.

[160] Windl W., Bunea M.M., Stumpf R., Dunham S.T., Masquelier M.P. (1999). First-Principles Study of Boron Diffusion in Silicon. Phys. Rev. Lett..

[161] Haddara Y.M., Folmer B.T., Law M.E., Buyuklimanli T. (2000). Accurate Measurements of the Intrinsic Diffusivities of Boron and Phosphorus in Silicon. Appl. Phys. Lett..

[162] Lide D.R. (2004). Handbook of Chemistry and Physics, 85th edition.

[163] Sugino O., Oshiyama A. (1992). Microscopic Mechanism of Atomic Diffusion in Si under Pressure. Phys. Rev. B.

[164] Khajavi L.T., Morita K., Yoshikawa T., Barati M. (2015). Thermodynamics of Boron Distribution in Solvent Refining of Silicon Using Ferrosilicon Alloys. J. Alloy. Compd..

[165] Beletskii A.K., Shcheretskii A.K., Vitusevich V.T., Shumikhin V.S. (1988). Enthalpies of Formation of Melts in the Si-B System. Izv. AN SSSR Met..

[166] Kudin V.G., Makara V.A., Sudavtsova V.S. (2001). Interactions in Molten Aluminum (Silicon)-Boron Alloys. Powder Metall. Met. Ceram..

[167] Franke P., Neuschütz D. (2004). B-Si. Binary systems. Part 2: Elements and Binary Systems from B-C to Cr-Zr.

[168] Kudin V.G., Makara V.A. (2002). Thermodynamic Properties of Metal-Boron Alloys. Inorg. Mater..

[169] Kaufman L., Uhrenius B., Birnie D., Taylor K. (1984). Coupled Pair Potential, Thermochemical and Phase Diagram Data for Transition Metal Binary Systems-VII. Calphad.

[170] Zaitseva N., Tsaplin A., Kodentsov A., Opila E.J., McNallan M.D. (2001). Thermodynamic Properties and Phase Equilibria in the Si-B System. High Temperature Corrosion and Materials Chemistry III.

[171] Gordienko S.P. (1996). Enthalpies of Formation for Boron Silicides. Powder Metall. Met. Ceram..

[172] George R.E., Witzel W., Riemann H., Abrosimov N.V., Nötzel N., Thewalt M.L.W., Morton J.J.L. (2010). Electron Spin Coherence and Electron Nuclear Double Resonance of Bi Donors in Natural Si. Phys. Rev. Lett..

[173] Sekiguchi T., Steger M., Saeedi K., Thewalt M.L.W., Riemann H., Abrosimov N.V., Nötzel N. (2010). Hyperfine Structure and Nuclear Hyperpolarization Observed in the Bound Exciton Luminescence of Bi Donors in Natural Si. Phys. Rev. Lett..

[174] Belli M., Fanciulli M., Abrosimov N.V. (2011). Pulse Electron Spin Resonance Investigation of Bismuth-Doped Silicon: Relaxation and Electron Spin Echo Envelope Modulation. Phys. Rev. B.

[175] Mohammady M.H., Morley G.W., Nazir A., Monteiro T.S. (2012). Analysis of Quantum Coherence in Bismuth-Doped Silicon: A System of Strongly Coupled Spin Qubits. Phys. Rev. B.

[176] Parry C.M. Bismuth-Doped Silicon: An Extrinsic Detector For Long-Wavelength Infrared (LWIR) Applications. Proceedings of SPIE 0244, Mosaic Focal Plane Methodologies.

[177] Williams R.S. (1907). On the Alloys of Antimony with Manganese, Chromium, Silicon and Tin; of Bismuth with Chromium and Silicon; and of Manganese with Tin and Lead. Z. Anorg. Chem..

[178] Girault B. (1977). Liquidus Curves of Some Metal-Silicon Systems. CR Hebd Sci. L Acad..

[179] Fuller C.S., Ditzenberger J.A. (1956). Diffusion of Donor and Acceptor Elements in Silicon. J. Appl. Phys..

[180] Ghoshtagore R.N. (1971). Donor Diffusion Dynamics in Silicon. Phys. Rev. B.

[181] Ishikawa Y., Yazaki K., Nakamichi I. (1989). The Diffusion of Bismuth in Silicon. Jpn. J. Appl. Phys..

[182] Smigelskas A.D., Kirkendall E.O. (1947). Zinc Diffusion in Alpha Brass. Trans. Aime.

[183] Kaban I., Gröbner J., Hoyer W., Schmid-Fetzer R. (2010). Liquid-liquid Phase Equilibria, Density Difference, and Interfacial Tension in the Al-Bi-Si Monotectic System. J. Mater. Sci..

[184] Scheel H.J. (2007). Introduction to Liquid Phase Epitaxy. Liquid Phase Epitaxy of Electronic, Optical and Optoelectronic Materials.

[185] Franke P., Neuschütz D. (2007). Ga-Si (Gallium-Silicon). Binary Systems. Part 5: Binary Systems Supplement 1.

[186] Klemm W., Klemm L., Hohmann E., Volk H., Orlamünder E., Klein H.A. (1948). Das Verhalten Der Elemente Der III. Gruppe Zueinander Und Zu Den Elementen Der IV. Gruppe. Z. Anorg. Chem..

[187] Savitskiy Y.M., Baron V.V., Tylkina M.A. (1958). Phase Diagrams on Properties of Alloys of Gallium and Thallium. Russ. J. Inorg. Chem..

[188] Keck P.H., Broder J. (1953). The Solubility of Silicon and Germanium in Gallium and Indium. Phys. Rev..

[189] Banerjee A. (1996). Advances in Physical Metallurgy.

[190] Haridoss S., Bénière F., Gauneau M., Rupert A. (1980). Diffusion of Gallium in Silicon. J. Appl. Phys..

[191] Kurtz A.D., Gravel C.L. (1958). Diffusion of Gallium in Silicon. J. Appl. Phys..

[192] Boltaks B.I., Dzhafarov T.D. (1964). Diffusion of Gallium in Inhomogeneous Silicon. Sov. Phys. Solid State.

[193] Kren J.G., Masters B.J., Wajda E.S. (1964). Effect of Surface Imperfections on Gallium Diffusion in Silicon. Appl. Phys. Lett..

[194] Makris J.S. (1971). Gallium Diffusions into Silicon and Boron-Doped Silicon. J. Appl. Phys..

[195] Kanibolotsky D.S., Golovata N.V., Bieloborodova O.A., Lisnyak V.V. (2003). Calorimetric Investigation of Liquid Gallium-Based Alloys. Z. Naturforsch. A.

[196] Sudavtsova V.S., Zinevich T.N., Kotova N.V., Beloborodova E.A. Thermodynamic Properties of Ga-Si (Ge, Sn, Pb) Melts. Russ. J. Phys. Chem..

[197] Tmar M., Pasturel A., Colinet C. (1983). Thermodynamics of (Silicon + Indium) and (Silicon + Gallium) Calorimetric Determination of the Partial Molar Enthalpy at Infinite Dilution of Si in Indium and Gallium. J. Chem. Thermodyn..

[198] Backenstoss G. (1957). Conductivity Mobilities of Electrons and Holes in Heavily Doped Silicon. Phys. Rev..

[199] Jones C.E., Schafer D.E., Scott M.W., Hager R.J. (1980). Studies of Indium-Doped Silicon.

[200] Cerofolini G.F., Ferla G., Pignatel G.U., Riva F. (1983). Thermodynamic and Kinetic Properties of Indium-Implanted Silicon II: High Temperature Diffusion in an Inert Atmosphere. Thin Solid Films.

[201] Millea M.F. (1966). The Effect of Heavy Doping on the Diffusion of Impurities in Silicon. J. Phys. Chem. Solids.

[202] Antoniadis D.A., Moskowitz I. (1982). Diffusion of Indium in Silicon Inert and Oxidizing Ambients. J. Appl. Phys..

[203] Noël J.-P., Hirashita N., Markert L.C., Kim Y.-W., Greene J.E., Knall J., Ni W.-X., Hasan M.A., Sundgren J.-E. (1989). Electrical Properties of Si Films Doped with 200-eV In^+^ Ions during Growth by Molecular-Beam Epitaxy. J. Appl. Phys..

[204] Scott W., Hager R.J. (1979). Solution Growth of Indium-Doped Silicon. J. Electron. Mater..

[205] Sato A., Suzuki K., Horie H., Sugii T. Determination of Solid Solubility Limit of In and Sb in Si Using Bonded Silicon-on-Insulator (SOI) Substrate. Proceedings of the IEEE International Conference on Microelectronic Test Structures (ICMTS).

[206] Solmi S., Parisini A., Bersani M., Giubertoni D., Soncini V., Carnevale G., Benvenuti A., Marmiroli A. (2002). Investigation on Indium Diffusion in Silicon. J. Appl. Phys..

[207] Liu J., Jeong U., Mehta S., Sherbondy J., Lo A., Shim K.H., Lim J.E., Ryssel H., Frey L., Gyulai J., Glawischnig H. (2000). Investigation of Indium Activation by C-V Measurement, in: Ion Implantation Technology.

[208] Yoshikawa T., Morita K., Kawanishi S., Tanaka T. (2010). Thermodynamics of Impurity Elements in Solid Silicon. J. Alloy. Compd..

[209] Cerofolini G.F., Ferla G., Pignatel G.U., Riva F., Ottaviani G. (1983). Thermodynamic and Kinetic Properties of Indium-Implanted Silicon I: Moderate Temperature Recovery of the Implant Damage and Metastability Effects. Thin Solid Films.

[210] Franke P., Neuschütz D. (2006). In-Si. Binary Systems. Part 3: Binary Systems from Cs-K to Mg-Zr.

[211] Pavlov P.V., Zorin E.I., Tetelbaum D.I., Khokhlov A.F. (1976). Nitrogen as Dopant in Silicon and Germanium. Phys. Status Solidi.

[212] Kaiser W., Thurmond C.D. (1959). Nitrogen in Silicon. J. Appl. Phys..

[213] Wu J., Sun J., Zhong X., Zhou Z., Wu C., Li F. (1999). Silicon Nitride Films Synthesized by Reactive Pulsed Laser Deposition in an Electron Cyclotron Resonance Nitrogen Plasma. Thin Solid Films.

[214] Ma X., Li C., Wang F., Zhang W. (2003). Thermodynamic Assessment of the Si-N System. Calphad.

[215] Blegen K. (1976). Equilibria and Kinetics in Systems Si-N, Si-O-N and Si-C-O-N. Ph.D. Thesis.

[216] Pehlke R.D., Elliott J.F. (1959). High-Temperature Thermodynamics of the Silicon, Nitrogen, Silicon-Nitride System. Trans. Am. Inst. Min. Metall. Eng..

[217] Guzman I.Y., Demidenko A.F., Koshchenko V.I., Fraifel’d M.S., Egner Y.V. (1976). Specific-Heats and Thermodynamic Functions of Si_3_N_4_ and Si_2_ON_2_. Izv. Akad. Nauk SSSR Neorg. Mater..

[218] Koshchenko V.I., Grinburg Y. (1985). Thermodynamic Functions of B6As (5–600 K), Beta-SiC (5–2500 K) and Si3N4 (5–4000 K). Izv. Akad. Nauk SSSR Neorg. Mater..

[219] Hillert M., Jonsson S., Sundman B. (1992). Thermodynamic Calculation of the Si-N-O System. Z. Met..

[220] Kaufman L. (1979). Calculation of Quasi Binary and Quasiternary Oxynitride Systems—III. Calphad.

[221] Dorner P., Gauckler L.J., Krieg H., Lukas H.L., Petzow G., Weiss J. (1981). Calculation of Heterogeneous Phase Equilibria in the SiAlON System. J. Mater. Sci..

[222] Hincke W.B., Brantley L.R. (1930). The High-Temperature Equilibrium Between Silicon Nitride, Silicon and Nitrogen. J. Am. Chem. Soc..

[223] Forgeng W.D., Decker B.F. (1958). Nitrides of Silicon. Min. Metall. Eng..

[224] Fujita N., Jones R., Goss J.P., Briddon P.R., Frauenheim T., Öberg S. (2005). Diffusion of Nitrogen in Silicon. Appl. Phys. Lett..

[225] Alpass C.R., Murphy J.D., Falster R.J., Wilshaw P.R. (2009). Nitrogen in Silicon: Diffusion at 500–750 °C and Interaction with Dislocations. Mater. Sci. Eng. B.

[226] Jones R., Hahn I., Goss J.P., Briddon P.R., Öberg S. (2004). Structure and Electronic Properties of Nitrogen Defects in Silicon. Solid State Phenom..

[227] Leslie W.C., Carroll K., Fishe R.M. (1952). Diffraction Patterns and Crystal Structures of Si_3_N_4_ and Ge_3_N_4_. Trans. Met. Soc. AIME.

[228] Carlson O.N. (1990). The N-Si (Nitrogen-Silicon) System. Bull. Alloy Phase Diagr..

[229] Arrowsmith J.M. (1963). A New Silicon Nitride Phase in Commercial Silicon Killed Steels. J. Iron Steel Inst..

[230] Mellor J.W. (1928). A Comprehensive Treatise on Inorganic and Theoretical Chemistry.

[231] Hengge E. (1962). Uber Die Darstellung Eines Neuen Siliciumsubnitrides (Si_6_N_2_)_n_. Z. Anorg. Allg. Chem..

[232] Wiberg E., Michaud H. (1954). Zur Kenntnis Eines Siliciumtetrazids Si(N_3_)_4_. Z. Naturforsch. Sect. B J. Chem. Sci..

[233] Peng H. (2004). Spark Plasma Sintering of Si3N4-Based Ceramics-Sintering Mechanism-Tailoring Microstructure-Evaluating Properties.

[234] Riedel R., Zerr A., Miehe G., Serghiou G., Schwarz M., Kroke E., Fueß H., Kroll P., Boehler R. (1999). Synthesis of Cubic Silicon Nitride. Nature.

[235] Wang C.-M., Pan X., Ruhle M., Riley F.L., Mitomo M. (1996). Silicon Nitride Crystal Structure and Observations of Lattice Defects. J. Mater. Sci..

[236] Scientific Group Thermodata Europe (1994). SGTE Substance Database.

[237] Dinsdale A.T. (1991). SGTE Data for Pure Elements. Calphad.

[238] Favre S., Nuta I., Chichignoud G., Zaïdat K., Chatillon C. (2016). Removing Phosphorus from Molten Silicon: A Thermodynamic Evaluation of Distillation. ECS J. Solid State Sci. Technol..

[239] Miki T., Morita K., Sano N. (1996). Thermodynamics of Phosphorus in Molten Silicon. Metall. Mater. Trans. B.

[240] Christensen J.S. (2004). Dopant Diffusion in Si and SiGe.

[241] Giessen V.B., Vogel R. (1959). About the Silicon-Phosphorus System. Z. Met..

[242] Olesinski R.W., Kanani N., Abbaschian G.J. (1985). The P-Si (Phosphorus-Silicon) System. Bull. Alloy Phase Diagr..

[243] Kooi E. (1964). Formation and Composition of Surface Layers and Solubility Limits of Phosphorus During Diffusion in Silicon. J. Electrochem. Soc..

[244] Abrikosov N.K., Glazov V.M., Chen-Yuan L. (1962). Individual and Joint Solubilities of Aluminum and Phosphorus in Germanium and Silicon. Russ. J. Lnorg. Chem..

[245] Uda K., Kamoshida M. (1977). Annealing Characteristics of Highly P^+^-Ion-Implanted Silicon Crystal—Two-Step Anneal. J. Appl. Phys..

[246] Solmi S., Parisini A., Angelucci R., Armigliato A., Nobili D., Moro L. (1996). Dopant and Carrier Concentration in Si in Equilibrium with Monoclinic SiP Precipitates. Phys. Rev. B.

[247] Yoshida M., Arai E., Nakamura H., Terunuma Y. (1974). Excess Vacancy Generation Mechanism at Phosphorous Diffusion into Silicon. J. Appl. Phys..

[248] Tsai J.C.C. (1969). Shallow Phosphorus Diffusion Profiles. IEEE.

[249] Tamura M. (1977). Dislocation Networks in Phosphorus-Implanted Silicon. Philos. Mag..

[250] Fogarassy E., Stuck R., Muller J.C., Grob A., Grob J.J., Siffert P. (1980). Effects of Laser Irradiation on Phosphorus Diffused Layers in Silicon. J. Electron. Mater..

[251] Mackintosh I.M. (1962). The Diffusion of Phosphorus in Silicon. J. Electrochem. Soc..

[252] Itoh K., Sasaki Y., Mitsuishi T., Miyao M., Tamura M. (1982). Thermal Behavior of B, P and As Atoms in Supersaturated Si Produced by Ion Implantation and Pulsed-Laser Annealing. Jpn. J. Appl. Phys..

[253] Nobili D., Armigliato A., Finnetti M., Solmi S. (1982). Precipitation as the Phenomenon Responsible for the Electrically Inactive Phosphorus in Silicon. J. Appl. Phys..

[254] Boeisenko V.E., Yudin S.G. (1987). Steady-State Solubility of Substitutional Impurities in Silicon. Phys. Status Solidi.

[255] Safarian J., Tangstad M. (2011). Phase Diagram Study of the Si-P System in Si-Rich Region. J. Mater. Res..

[256] Jung I.-H., Zhang Y. (2012). Thermodynamic Calculations for the Dephosphorization of Silicon Using Molten Slag. JOM.

[257] Liang S.-M., Schmid-Fetzer R. (2014). Modeling of Thermodynamic Properties and Phase Equilibria of the Si-P System. J. Phase Equilib. Diffus..

[258] Fritz G., Berkenhoff H.O. (1959). Uber Ein Siliciumphosphid Si_2_P. Z. Anorg. Allg. Chem..

[259] Wadsten T. (1975). Preparative and Crystal-Structure Studies on Orthorhombic Silicon Monophosphide. Chem. Scr..

[260] SpringThorpe A.J. (1969). The Preparation of Single Crystal Orthorhombic SiP_2_. Mater. Res. Bull..

[261] Ntep J.-M., Said Hassani S., Lusson A., Tromson-Carli A., Ballutaud D., Didier G., Triboulet R. (1999). ZnO Growth by Chemical Vapour Transport. J. Cryst. Growth.

[262] Carlsson J.R.A. (1997). A New Silicon Phosphide, Si_12_P_5_: Formation Conditions, Structure, and Properties. J. Vac. Sci. Technol. A Vac. Surf. Films.

[263] Zaitsev A.I., Shelkova N.E., Kodentsov A.A. (2000). Thermodynamic Properties and Phase Equilibria in the Silicon-Phosphorous System. J. Phase Equilib..

[264] Gurvich L.V. (1983). Ivtantermo-Automatic Data System on Thermodynamic Properties of Substances. Vestn. Akad. Nauk SSSR.

[265] The Japan Society for Promotion of Science (1988). The 19th Committee in Steelmaking: Steelmaking Data Sourcebook.

[266] Pelton A.D., Degterov S.A., Eriksson G., Robelin C., Dessureault Y. (2000). The Modified Quasichemical Model I—Binary Solutions. Metall. Mater. Trans. B.

[267] Hillert M. (2001). The Compound Energy Formalism. J. Alloy. Compd..

[268] Ugai Y.A., Sokolov L.I., Goncharov E.G., Makarov V.S. (1987). PTx Composition Diagram and Thermodynamics of Phase Equilibrium in the Silicon-Phosphorus System. Russ. J. Inorg. Chem..

[269] Knacke O., Kubaschewski O., Hesselmann K. (1991). Thermochemical Properties of Inorganic Substances.

[270] Philipp F., Schmidt P. (2008). The Cationic Clathrate Si_46−2x_P_2x_Te_x_ Crystal Growth by Chemical Vapour Transport. J. Cryst. Growth.

[271] Wilmsen C.W., Wilmsen C.W. (1985). Physics and Chemistry of III-V Compound Semiconductor Interfaces.

[272] Arutyunyan N.A., Zaitsev A.I., Shaposhnikov N.G. (2011). Analysis of Thermodynamic Properties and Phase Equilibria in the Si-P System. Russ. J. Phys. Chem. A.

[273] Knudsen M. (1909). Die Gesetze Der Molekularströmung Und Der Inneren Reibungsströmung Der Gase Durch Röhren. Ann. Phys..

[274] Olesinski R.W., Abbaschian G.J. (1985). The Sb-Si (Antimony-Silicon) System. Bull. Alloy Phase Diagr..

[275] Malmeja Y., Desré P., Bonnier E. (1972). Contribution to the Ternary Phase Diagram of Ge-Si-Sb. Mém. Sci. Rev. Métall. Fr..

[276] Rohan J.J., Pickering N.E., Kennedy J. (1959). Diffusion of Radioactive Antimony in Silicon. J. Electrochem. Soc..

[277] Wang J., Liu Y.J., Liu L.B., Zhou H.Y., Jin Z.P. (2011). Thermodynamic Modeling of the Au-Sb-Si Ternary System. J. Alloy. Compd..

[278] Nobili D. (1989). Equilibrium Carrier Density and Solubility of Antimony in Silicon. J. Electrochem. Soc..

[279] Zhao B., Zhou J., Chen Y. (2010). Numerical Simulation of the Impurity Photovoltaic Effect in Silicon Solar Cells Doped with Thallium. Phys. B Condens. Matter.

[280] Tamaru S. (1909). Metallographische Mitteilungen Aus Dem Institut Für Physikalische Chemie Der Universität Göttingen. LXIX. Über Die Legierungen Des Siliciums Mit Zinn, Blei Und Thallium. Z. Anorg. Chem..

[281] Predel B., Madelung O. (1985). Si-Tl (Silicon-Thallium). Pu-Re—Zn-Zr.

[282] Schmit J.L., Scott M.W. (1981). Growth of Thallium-Doped Silicon from a Tin-Thallium Solution. U.S. Patent.

[283] Ghoshtagore R.N. (1971). Dopant Diffusion in Silicon. III. Acceptors. Phys. Rev. B.

[284] Burton J.A., Kolb E.D., Slichter W.P., Struthers J.D. (1953). Distribution of Solute in Crystals Grown from the Melt. Part II. Experimental. J. Chem. Phys..

[285] Cordero B., Gómez V., Platero-Prats A.E., Revés M., Echeverría J., Cremades E., Barragán F., Alvarez S. (2008). Covalent Radii Revisited. Dalton Trans..

